# The Transcriptomic Response of Rat Hepatic Stellate Cells to Endotoxin: Implications for Hepatic Inflammation and Immune Regulation

**DOI:** 10.1371/journal.pone.0082159

**Published:** 2013-12-09

**Authors:** Stephen A. K. Harvey, Anil Dangi, Ashish Tandon, Chandrashekhar R. Gandhi

**Affiliations:** 1 Department of Opthalmology, University of Pittsburgh, Pittsburgh, Pennsylvania, United States of America; 2 Department of Surgery, University of Cincinnati, Cincinnati, Ohio, United States of America; 3 Cincinnati VA Medical Center, Cincinnati, Ohio, United States of America; 4 Cincinnati Children’s Hospital Medical Center, Cincinnati, Ohio, United States of America; French National Centre for Scientific Research, France

## Abstract

With their location in the perisinusoidal space of Disse, hepatic stellate cells (HSCs) communicate with all of the liver cell types both by physical association (cell body as well as cytosolic processes penetrating into sinusoids through the endothelial fenestrations) and by producing several cytokines and chemokines. Bacterial lipopolysaccharide (LPS), circulating levels of which are elevated in liver diseases and transplantation, stimulates HSCs to produce increased amounts of cytokines and chemokines. Although recent research provides strong evidence for the role of HSCs in hepatic inflammation and immune regulation, the number of HSC-elaborated inflammatory and immune regulatory molecules may be much greater then known at the present time. Here we report time-dependent changes in the gene expression profile of inflammatory and immune-regulatory molecules in LPS-stimulated rat HSCs, and their validation by biochemical analyses. LPS strongly up-regulated LPS-response elements (TLR2 and TLR7) but did not affect TLR4 and down-regulated TLR9. LPS also up-regulated genes in the MAPK, NFκB, STAT, SOCS, IRAK and interferon signaling pathways, numerous CC and CXC chemokines and IL17F. Interestingly, LPS modulated genes related to TGFβ and HSC activation in a manner that would limit their activation and fibrogenic activity. The data indicate that LPS-stimulated HSCs become a major cell type in regulating hepatic inflammatory and immunological responses by altering expression of numerous relevant genes, and thus play a prominent role in hepatic pathophysiology including liver diseases and transplantation.

## Introduction

The liver presents the first line of host defense against pathogens, toxins and aberrant cells by removing them from the portal circulation. It contains phagocytic Kupffer cells (KCs) as well as immune competent cells, including antigen-presenting cells (APCs) [dendritic cells (DCs) and KCs], natural killer (NK) and NKT cells, and T cells [1-3]. DCs and KCs activate effector T cells in an antigen-specific and MHC-restricted manner, which elicit immune response. The liver exhibits immune tolerance as evident from its harboring of viruses (HBV and HCV) and infectious organisms (e.g., malaria parasite), portal venous and oral tolerance to antigens, and tumor metastasis [4,5], as well as the maintenance of the allograft function in some liver transplant recipients who discontinue immunosuppressive drugs, and in many animal models across the MHC barriers [6-12]. Furthermore, the early phase of transplantation is characterized by inflammatory and ischemia/reperfusion-induced liver injury, which is repaired within a reasonable time period. During exposure to various pathogens and toxins as well as physical trauma also the liver is subjected to inflammatory injury that must be resolved in a timely manner to regain physiologic structure and function. Recent evidence indicates that the perisinusoidal hepatic stellate cells (HSCs) play an important role in the regulation of hepatic inflammation and immunity [13].

HSCs, a major site of retinoid(s) storage and quiescent physiologically, become activated progressively during liver injury by releasing retinoids and acquiring a myofibroblast-like fibrogenic, contractile and proliferative phenotype. Such progressive activation of HSCs can be achieved in cell culture from quiescent [high retinoids and no α-smooth muscle actin (α-sma) expression] through transitionally activated (moderate retinoids and α-sma expression) to highly activated (low or no retinoids and high α-sma expression) [13]. Transitionally activated HSCs are found in the liver during acute liver injury and early times after transplantation. We had hypothesized that interactions of HSCs with the gram-negative bacterial endotoxin (lipopolysaccharide, LPS), produced in the gut and delivered to the liver via portal vein almost continuously, might have significant influence on the hepatic inflammatory and immune responses. We found that both quiescent [14] and transitionally activated [15-17] rat HSCs respond to very low levels of LPS (1 ng/ml), and produce nitric oxide (NO), tumor necrosis factor α (TNFα) and interleukin 6 (IL6). Mouse HSCs were also found to produce these and several other cytokines and chemokines, synthesis of which is influenced by LPS [18-20]. 

Both human [21] and murine HSCs [19,20] produce chemokines that induce chemotaxis of conventional and regulatory T cells (Tregs) and DCs; also, HSCs’ large stores of retinoic acid and ability to produce TGFβ can potentially influence the homing of migratory immune cells [22]. However, relative to gut DCs, HSCs exhibited much lower potential to induce CCR9 and α4β7 expression on CD8 T cells, and even the addition of all-trans retinoic acid failed to increase this effect [23]. HSCs were shown to present lipid and peptide antigens to NKT and CD4/CD8 T cells respectively [24], but they also induce apoptosis of CD4 and CD8 T cells [19,25], and inhibit splenic DC-induced proliferation of CD8 T cells in a CD54-dependent manner [26] and CD3/CD28-induced activation/proliferation of CD8 T cells by expressing B7H4 [27]. In contrast, HSCs promote expansion of Tregs [19,28] that is augmented when HSCs are pre-treated with LPS [19], enhance DC- and TGFβ-mediated expansion of Tregs, and block TGFβ-induced differentiation of T helper 17 (Th17) cells [29]. Interestingly, HSC-modulated Tregs possess greater immunosuppressive potential than control cells [19], and HSC-conditioned DCs exhibit reduced potential to activate T cells [20]. We recently reported decreased LPS-induced hepatic infiltration of neutrophils in mice depleted of HSCs [30]. Although this information indicates critical role of HSCs in hepatic inflammation and immune regulation via cell membrane-associated and soluble factors, the complete repertoire of the mediators elaborated by LPS-stimulated HSCs is likely much larger than reported in the literature. To gain such understanding, which can be useful for future research, we determined time-dependent changes in inflammatory and immune-regulatory molecules in LPS-stimulated HSCs.

## Materials and Methods

### Isolation and culture of stellate cells

The experimental protocols were reviewed and approved by the Institutional Animal Care and Use Committees of the University of Pittsburgh (#1108658), University of Cincinnati (#12-10-16-01) and Veterans Administration at Cincinnati (12-08-17-02) in accordance with National Institutes of Health guidelines. HSCs were prepared from the livers of male Sprague-Dawley rats as described previously [14,17,19]. The cells were plated at a density of 0.5 x 10^6^ cells/cm^2^, and the medium renewed after overnight culture and then on alternate days. The cells were used on day 7 of culture when the majority expressed α-sma but contained abundant retinoids as assessed by immunohistochemistry, vitamin A autofluorescence and Oil Red staining indicative of transitionally activated phenotype [20]. The purity was also determined via flow and Western blot analysis to rule out contamination of Kupffer cells, endothelial cells and myeloid cells, as well as via immunohistochemistry for α-sma in conjunction with vitamin A autofluorescence (Figure S1).

### Microarray analysis of HSCs

In two independent experiments, rat HSCs were cultured with or without 10 ng/ml LPS for 1h and 24h, then harvested for microarray analysis (i.e., 8 samples). In the first of these experiments, cells were also harvested after 3h, 6h and 12h of LPS treatment, for a total of 11 samples. Total RNA was isolated using the Qiagen RNeasy/Qiashredder systems (Qiagen Inc., Valencia, CA), and processed and analyzed using the appropriate Affymetrix products (Affymetrix Inc., Santa Clara, CA: cited as catalog numbers in boxed parentheses). Eukaryote Poly A RNA internal standards [900433] were added to the samples, and the mRNA component of the total RNA was reverse-transcribed in the presence of a T7-(dT)24 primer [900431]. The resulting cDNA was extracted [900371] and transcribed in the presence of biotin-labeled ribonucleotides [900449]. 20 μg of the biotinylated RNA was fragmented [900371] for 35 minutes at 94°C. Each sample was hybridized overnight to a Rat Genome 230 2.0 Array [900506]. These arrays contained >31,000 panels, each targeting a specific transcript sequence. Approximately 13,400 gene products identified by Entrez Gene numbers are redundantly targeted by 20,500 panels; the remaining panels target products have not yet been assigned Entrez Gene numbers. Affymetrix GCOS 1.4 software was used to assess the presence or absence of the target sequence of each panel, and to make pairwise statistical comparisons among samples. The unscaled mean value was 279 ± 73 (mean ± SD, n = 11), and expression levels were scaled to 500 using the GCOS default method (2% trimmed mean). Across all samples, 46.0 ± 3.6% of panels detected their cognate transcript sequences; 11,379 target sequences were absent from every sample and were omitted from further consideration. In both experiments genes were selected if any LPS-treated time point showed a valid 2-fold change relative to the 1h control. A valid change required that the GCOS software called the higher expressing sample Present (i.e., cognate transcript detected) and that software comparison between the samples showed a significant increase or decrease. Samples treated with LPS for 24h were also compared against the 24h control. Pairwise comparisons of control vs stimulated samples yielded (2 control x 5 stimulated = 10 comparisons) for the first experiment and (2 control x 2 stimulated = 4 comparisons) for the second, a total of 14 pairwise comparisons. 

GCOS generated CEL files and a summary Excel spreadsheet have been posted in the NCBI GEO database:

http://www.ncbi.nlm.nih.gov/geo/query/acc.cgi?token=njqfbyueuowsalc&acc=GSE49980

The web-based Ingenuity Pathway Analysis software was used to assign responsive genes to known canonical pathways. 

### Cytokine and nitric oxide measurements

IL6, TNFα, IL1α, IL4, IL13 and RANTES (CCL5) were measured in HSC culture supernatants with a custom-made rat Multi-Analyte ELISArray kit (SA Biosciences). The concentration of nitrite and nitrate (NO end products) was determined using a colorimetric assay kit (Canyan Chemicals, Ann Arbor, MI).

### Quantitative real time-PCR (qPCR) and Western analysis

Expression levels of various mRNAs were measured via qPCR. Briefly, RNAs were prepared from the cells using TRIzol Reagent (Invitrogen), and cDNAs were prepared using high-capacity cDNA reverse transcription kit (Applied Biosystems). qRT-PCR was performed using the Sybr green master mix and 7300 Fast Real-Time PCR System (Applied Biosystems) with PCR primers listed in Table S1. 

 For protein (Western) analysis, cell lysates were prepared as described previously [14,17]. 20 µg of protein was subjected to SDS–PAGE, and the separated proteins transferred on to an Immobilon-P membrane (Millipore, Bedford, MA). After blocking non-specific binding and incubation with appropriate secondary antibody, detection was achieved using an ECL chemiluminescence kit (Amersham-Pharmacia). The Abs used were TLR4 (goat polyclonal: sc12511), MD2 (rabbit polyclonal: sc-20668) and CD14 (goat polyclonal: sc5749) (Santa Cruz Biotechnology, Inc., Santa Cruz, CA), Myd88 (rabbit polyclonal) (Imgenex, San Diego, CA), and α-sma (mouse monoclonal clone 1A4, A2547) (Sigma, St Louis, MO). 

## Results and Discussion

### Comparison of experiments 1 and 2

Comparison of the T1 and T24 responses between experiments (Table S2) showed that of the aggregate 2,065 genes responsive at either time in either experiment, 598 responded in both experiments. Of these 567 (95%) were concordant, responding in the same direction in both experiments while 31 genes (5%) were discordant, showing opposite directions of response. Canonical pathways analysis using IPA software gave the relative enrichment of pathways (as -log(p) values) for aggregate, concordant, and experiment-exclusive groups (Tables S3 and S4). Concordance better identified the 29 pathways in Table S2, where enrichment of the concordant group exceeded that of the aggregate group. For the 33 pathways in Table S3, the aggregate group has greater enrichment, so the pathways may have different member contributions from the different experiments. Notably, for very few pathways (7 of 62 total; values in bold in the Tables S3 and S4) is the enrichment of experiment-exclusive groups greater than the concordant group, indicating that no important pathways are exclusive to either of the individual experiments.

A full survey of concordance was made using contingency analysis (χ^2^ test, p < 10 ^-300^); this included genes which responded in the first experiment at intermediate times (3h, 6h or 12h) and were also modulated in the second experiment. (Figure S2). Genes with ≥ 3 of 10 valid 2-fold changes in the first experiment were more than twice as likely to be modulated in the second experiment, relative to random chance. This concordance was so marked that genes with ≥ 7 changes in the first experiment and ≥ 3 changes in the second were ≥ 9-fold more common than expected by chance, while genes with ≥ 7 changes in the first experiment and zero changes in the second appeared ≥ 2-fold **less** frequently would than expected. 

Combining the 7 LPS stimulated samples irrespective of time permits a nonparametric (Mann-Whitney rank sum) test against the 4 control samples, with p < 0.042 for control rank sums which are ≤ 13 or ≥ 35. The 10,903 unique characterized genes with n (14 ≥ n ≥ 0) valid 2-fold differences (Figure S3, unfilled bars) were examined for statistical significance. The 1,692 unique characterized genes with changes at p < 0.042 (filled bars) represent a progressively increasing fraction of the total changes for a given value of n (filled circles, right hand axis). Thus for genes that showed n ≥ 9 changes, those changes were predominantly (fraction > 0.5) statistically significant.

#### LPS-induced alterations in gene transcripts in HSCs

In addition to the time-dependent changes in HSCs due to LPS stimulation shown Figures 1-9, the magnitude and times of earliest and maximal changes occurring in the various transcripts are shown in Table 1 for a quick reference. The transcripts that are not altered are not shown in this Table.

**Figure 1:**
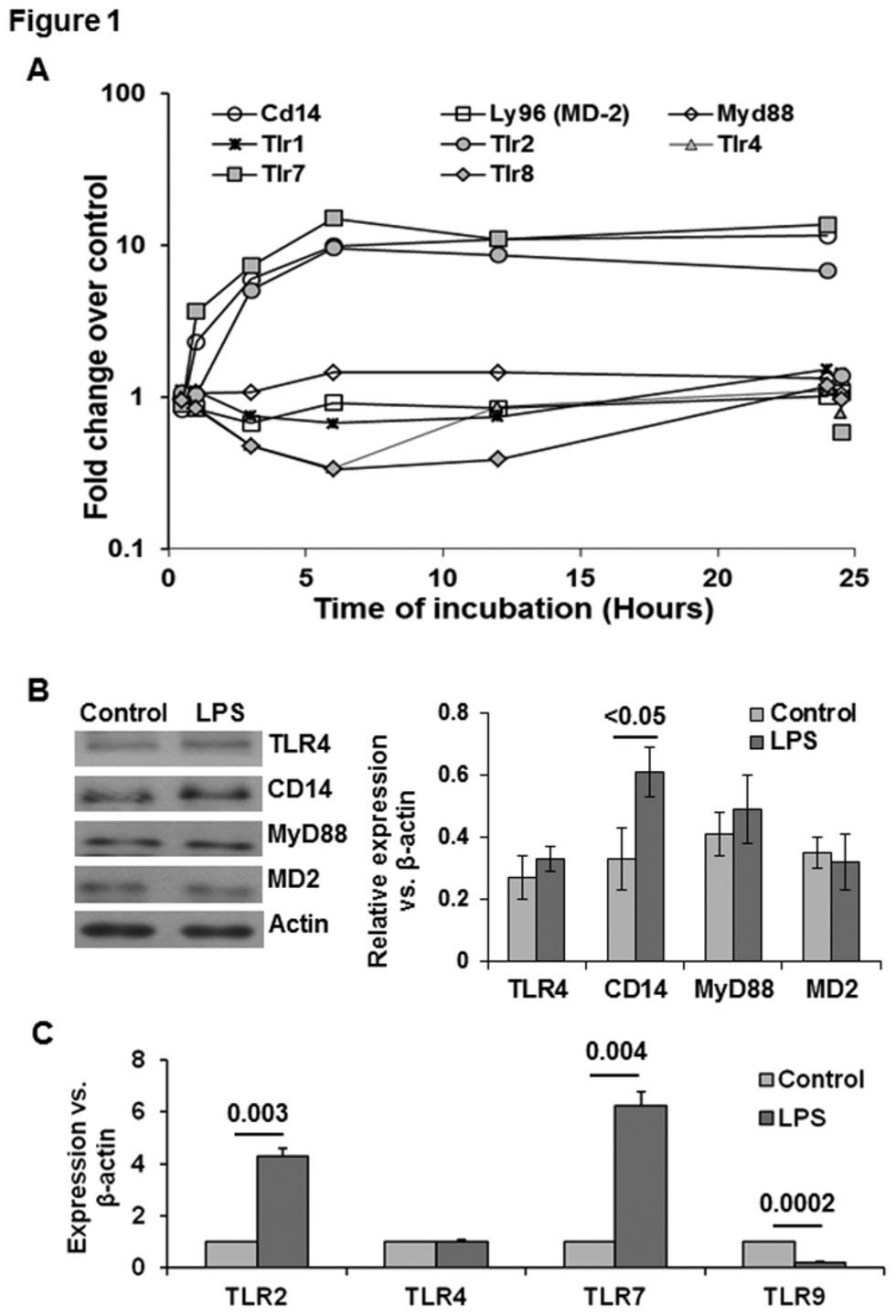
TLR and other LPS-response elements in LPS-stimulated HSCs. (A) Microarray data show time-dependent changes in the indicated transcripts. For clarity control gene expression at 1 and 24h is offset to 0.5h and 24.5h respectively. (B) A representative Western blot (left pane) and densitometric analysis (right panel) of the indicated molecules at 24h following stimulation with 10 ng/ml LPS. (C) qPCR analysis of the indicated molecules with p values showing statistical differences. The values (B,C) shown are form 3 separate determinations from different batches of HSCs. Statistical significance was derived from student’s t-test using Microsoft-excel program.

**Figure 2:**
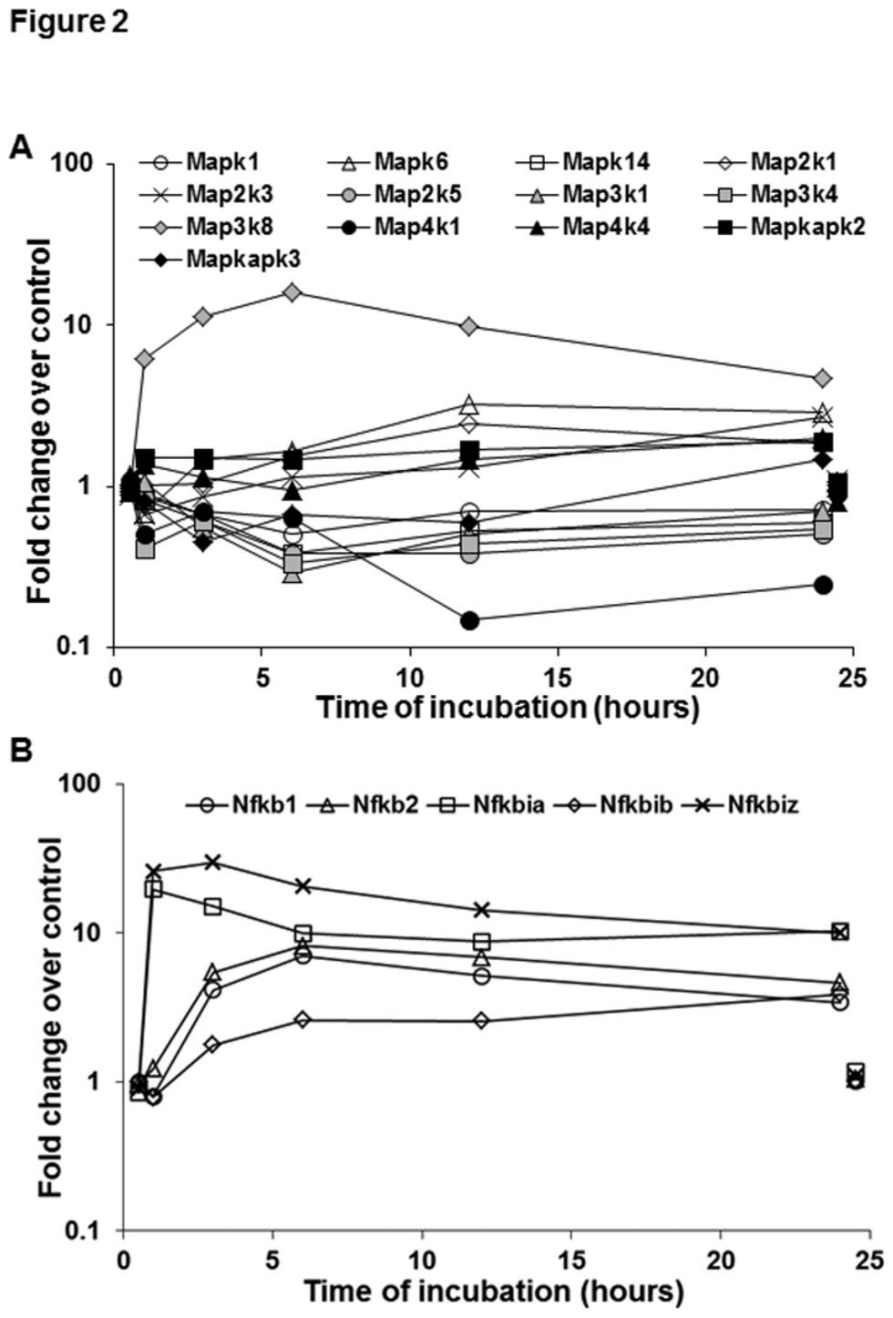
Gene expression of MAPKs and NFkB signaling in LPS-stimulated HSCs. Microarray data show changes in the indicated transcripts for MAPK related (A) and NFkB-related genes (B) in HSCs stimulated with 10 ng/ml LPS. For clarity control gene expression at 1 and 24h is offset to 0.5h and 24.5h respectively.

**Figure 3:**
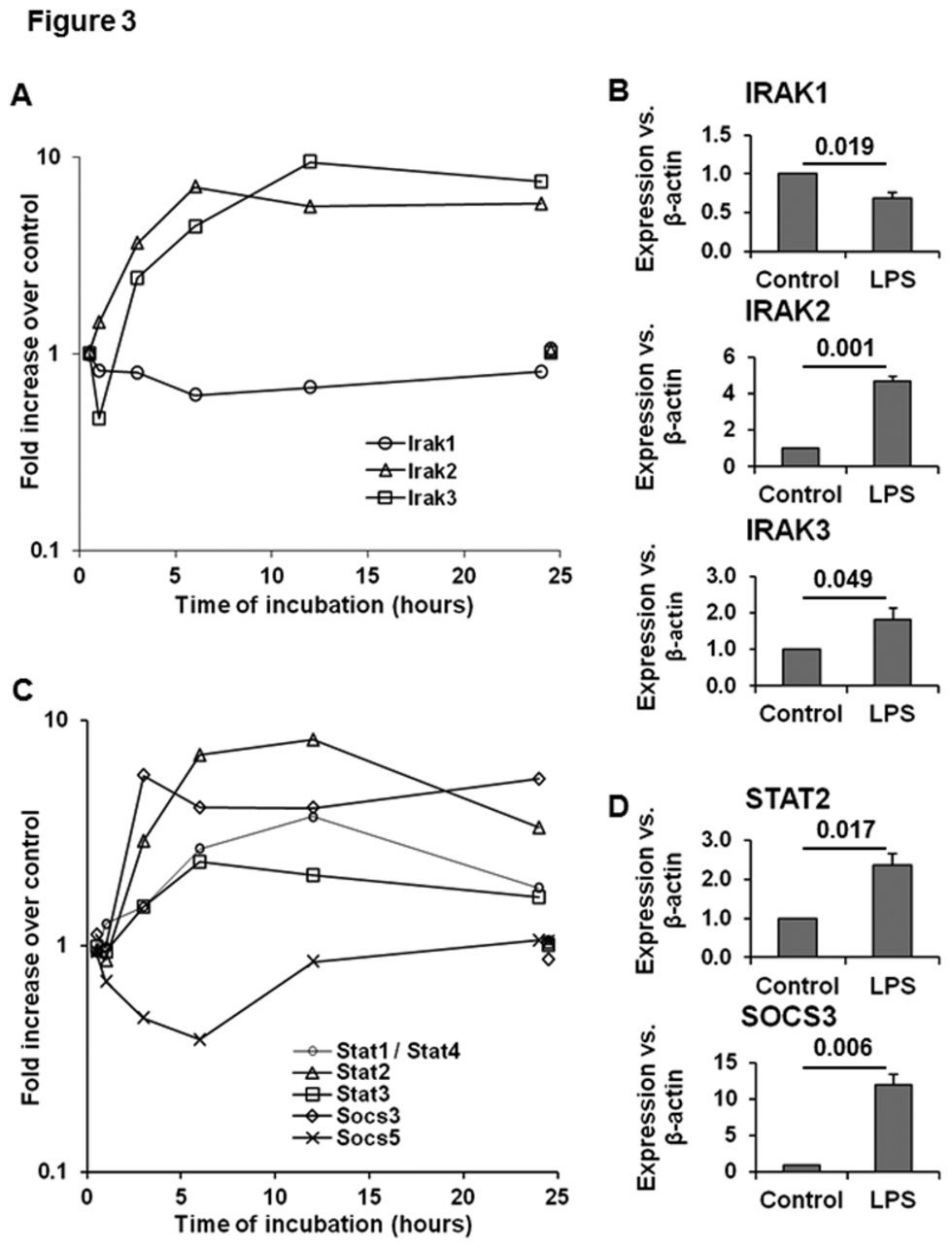
Gene expression of IRAK, STAT and SOCS signaling in LPS-stimulated HSCs. Microarray data show LPS-induced time-dependent changes in the transcripts for Irak1-3 (A), and Stat1-3, Socs3 and Socs5 (C), and their mRNA expressions as determined by qPCR at 24h following stimulation with LPS (B and D). For clarity control gene expression at 1 and 24h is offset to 0.5h and 24.5h respectively. The numbers in the histograms (B and D) are the p values form 3 separate determinations from different batches of HSCs. Statistical significance was derived from student’s t-test using Microsoft-excel program.

**Figure 4:**
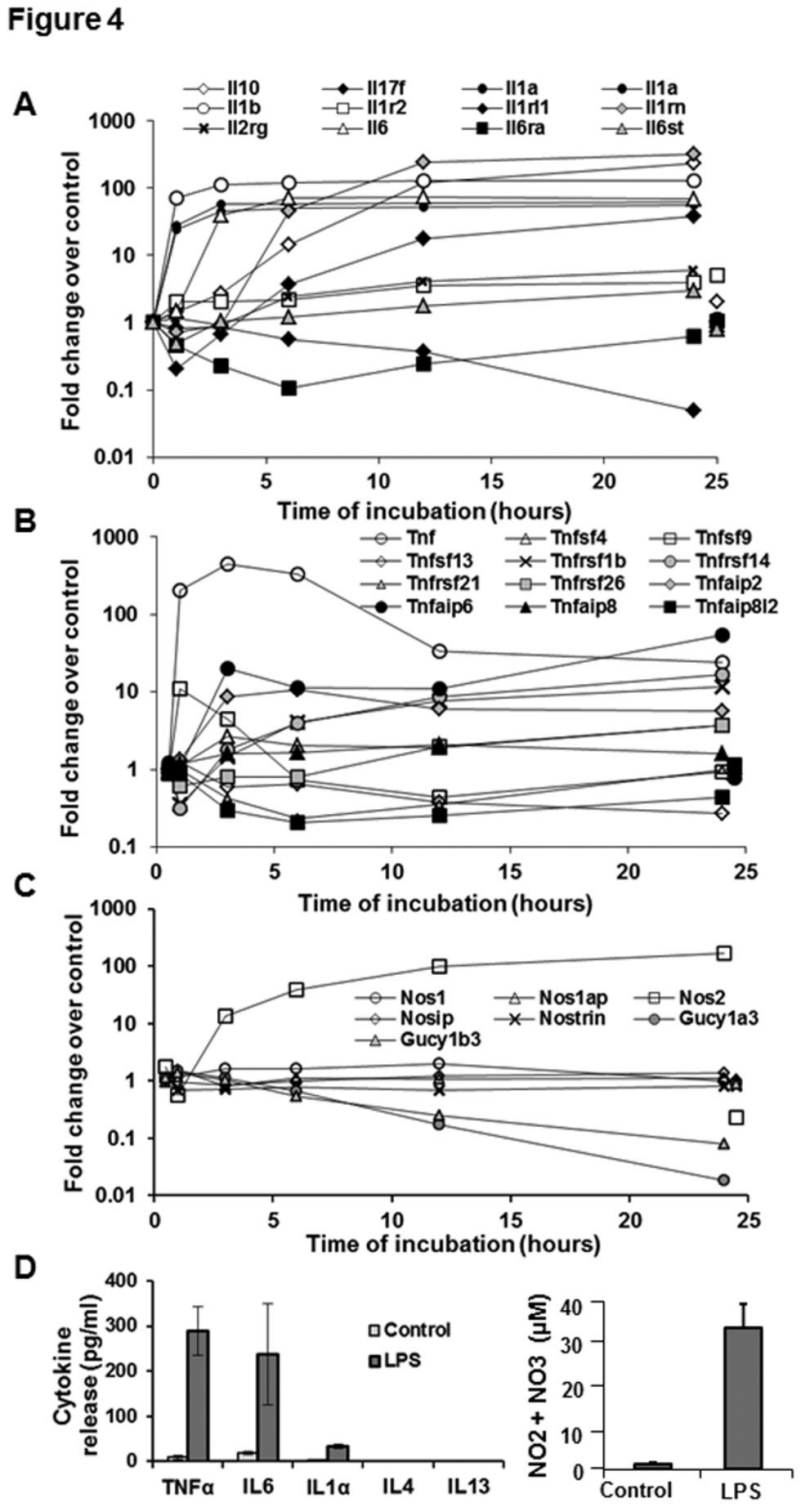
Changes in interleukins, TNF family members, iNOS and functionally related genes in LPS-stimulated HSCs. (A-C) Microarray data show changes in the indicated transcripts for interleulins, TNF-related genes and NOS-related genes. For clarity control gene expression at 1 and 24h is offset to 0.5h and 24.5h respectively. (D) Release of cytokines as measured by ELISA and of NO2+NO3 (a measure of NO synthesis) in the culture supernatants of unstimulated and LPS (10 ng/ml)-stimulated cells at 24h. The values form 3 separate determinations from different batches of HSCs. Statistical significance was derived from student’s *t*-test using Microsoft-excel program.

**Figure 5:**
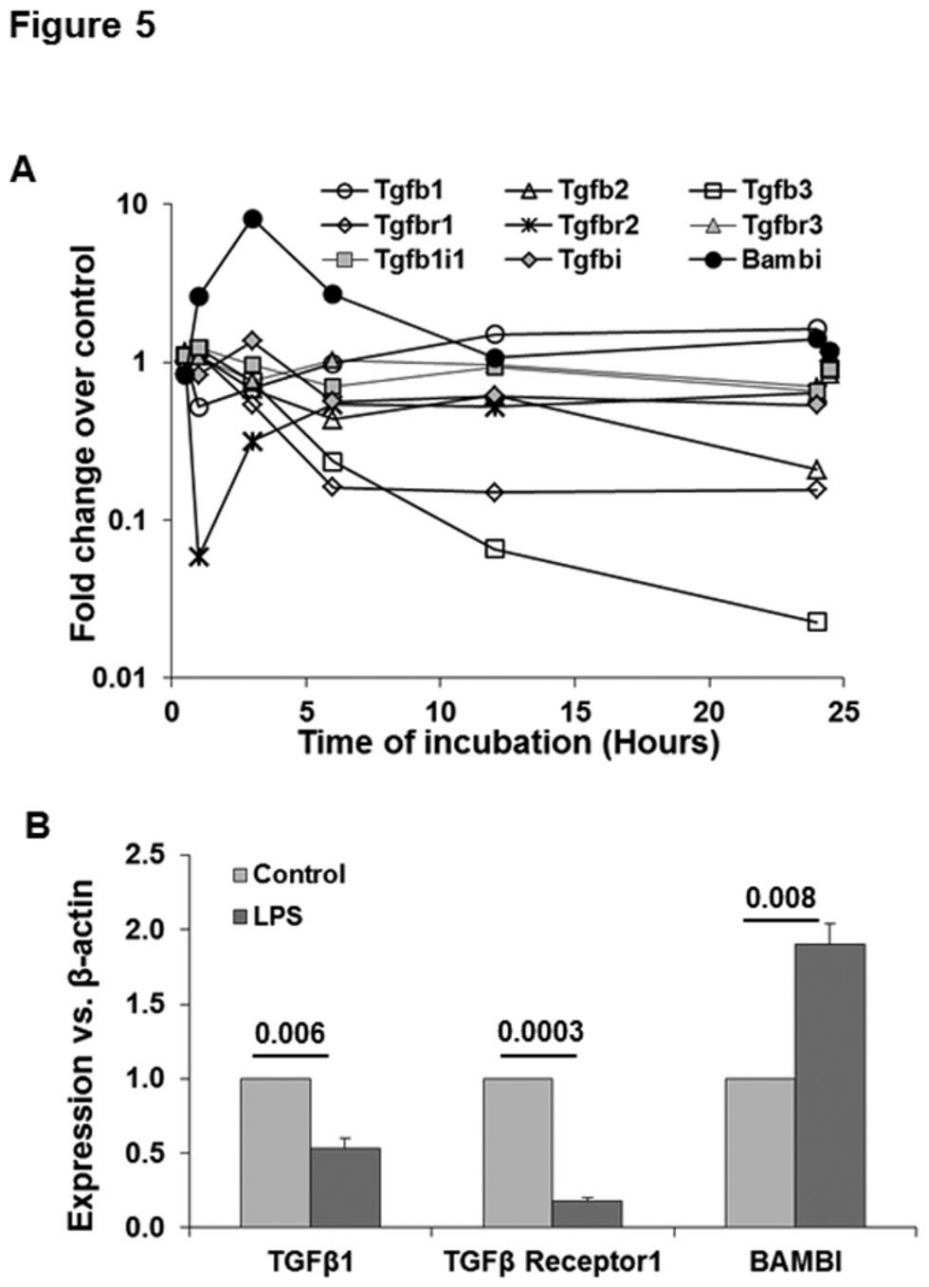
Changes in TGFβ and related genes in LPS-stimulated HSCs. (A) Microarray data show LPS-induced changes in the indicated transcripts in HSCs. For clarity control gene expression at 1 and 24h is offset to 0.5h and 24.5h respectively. (B) qPCR data showing mRNA expression of the indicated molecules at 24h following stimulation with 10 ng/ml LPS. The numbers are p values form 3 separate determinations from different batches of HSCs. Statistical significance was derived from student’s *t*-test using Microsoft-excel program.

**Figure 6:**
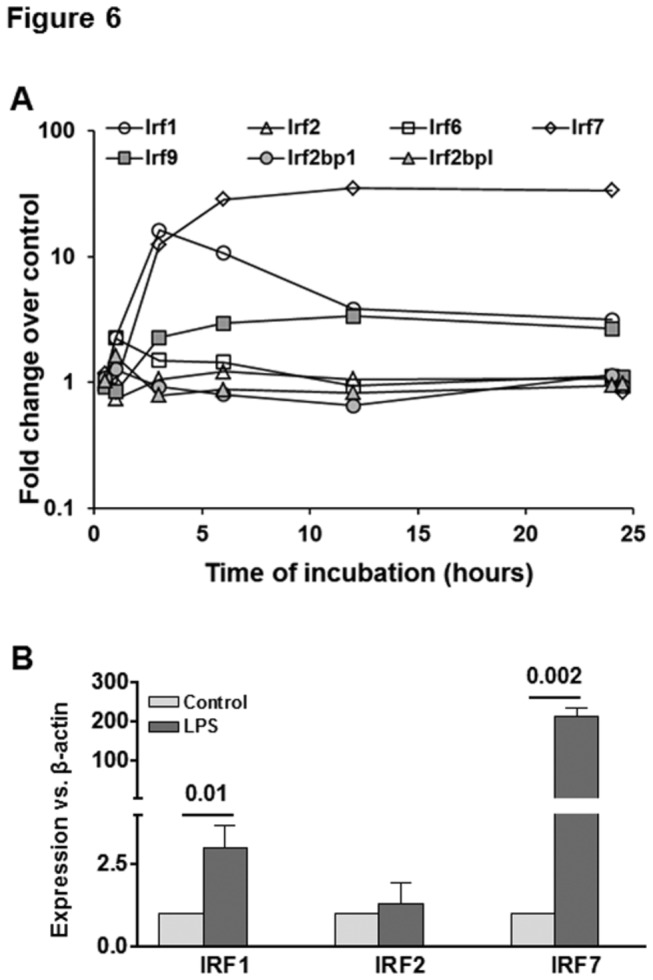
Changes in IRF and related genes in LPS-stimulated HSCs. (A) Microarray data show LPS-induced in the indicated transcripts. For clarity control gene expression at 1 and 24h is offset to 0.5h and 24.5h respectively. (B) qPCR data showing mRNA expression of the indicated molecules at 24h following stimulation with 10 ng/ml LPS. The numbers are p values form 3 separate determinations from different batches of HSCs. Statistical significance was derived from student’s *t*-test using Microsoft-excel program.

**Figure 7:**
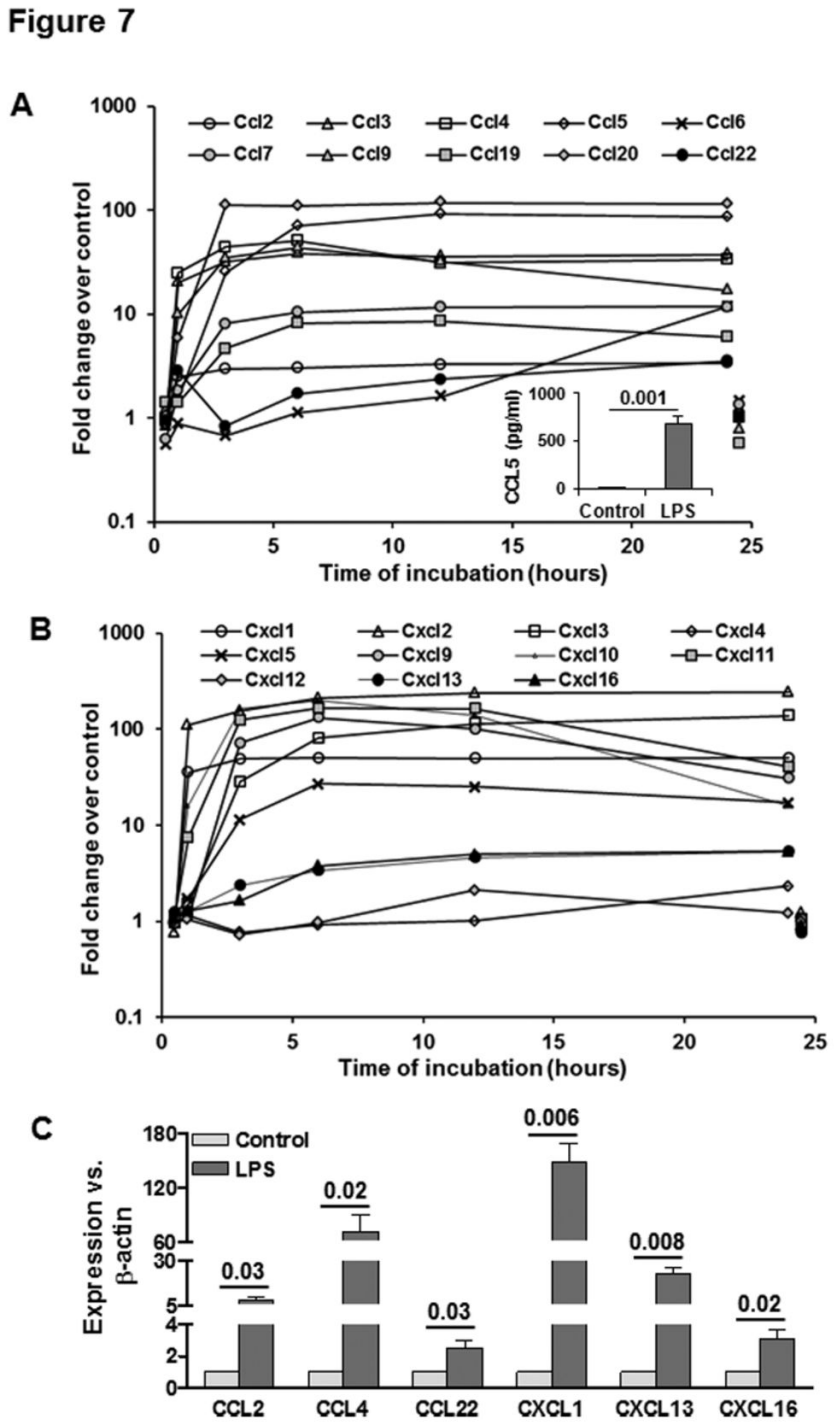
LPS-induced changes in Ccl and Cxcl chemokines. Microarray data show LPS-induced changes in CCL (A) and CXCL (B) class of chemokines in HSCs. For clarity control gene expression at 1 and 24h is offset to 0.5h and 24.5h respectively. Inset in (A) shows CCL5 release by HSCs stimulated with 10 ng/ml LPS for 24h as determined by ELISA. (C) qPCR data showing mRNA expression of the indicated molecules at 24h following stimulation with 10 ng/ml LPS. The numbers are p values form 3 separate determinations from different batches of HSCs. Statistical significance was derived from student’s *t*-test using Microsoft-excel program.

**Figure 8:**
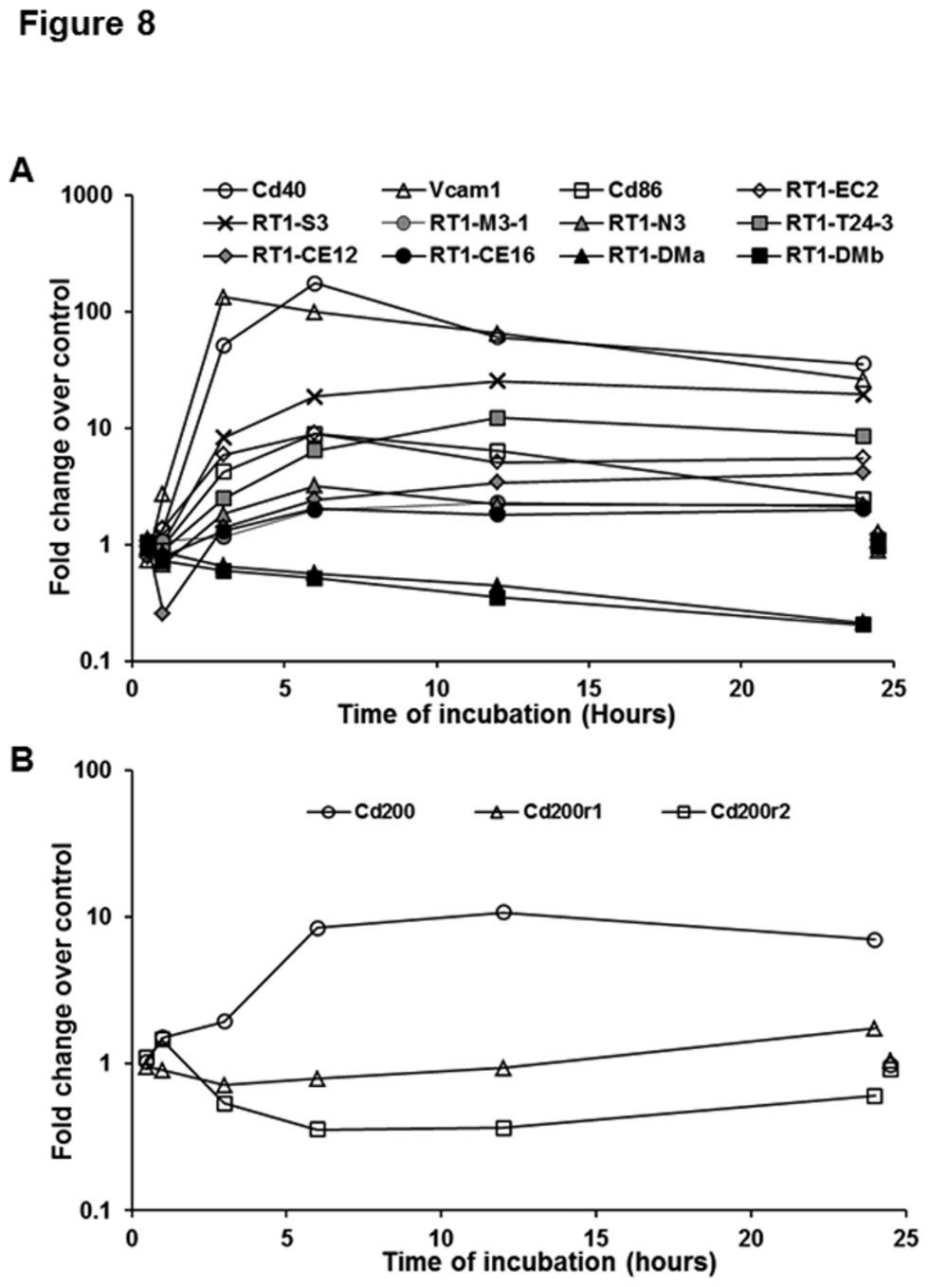
LPS-induced changes in antigen-presenting molecules. Microarray data show LPS-induced changes in the transcripts for indicated antigen-presenting and co-modulatory molecules (A), and for CD200 and its receptors (B) in HSCs. For clarity control gene expression at 1 and 24h is offset to 0.5h and 24.5h respectively.

**Figure 9:**
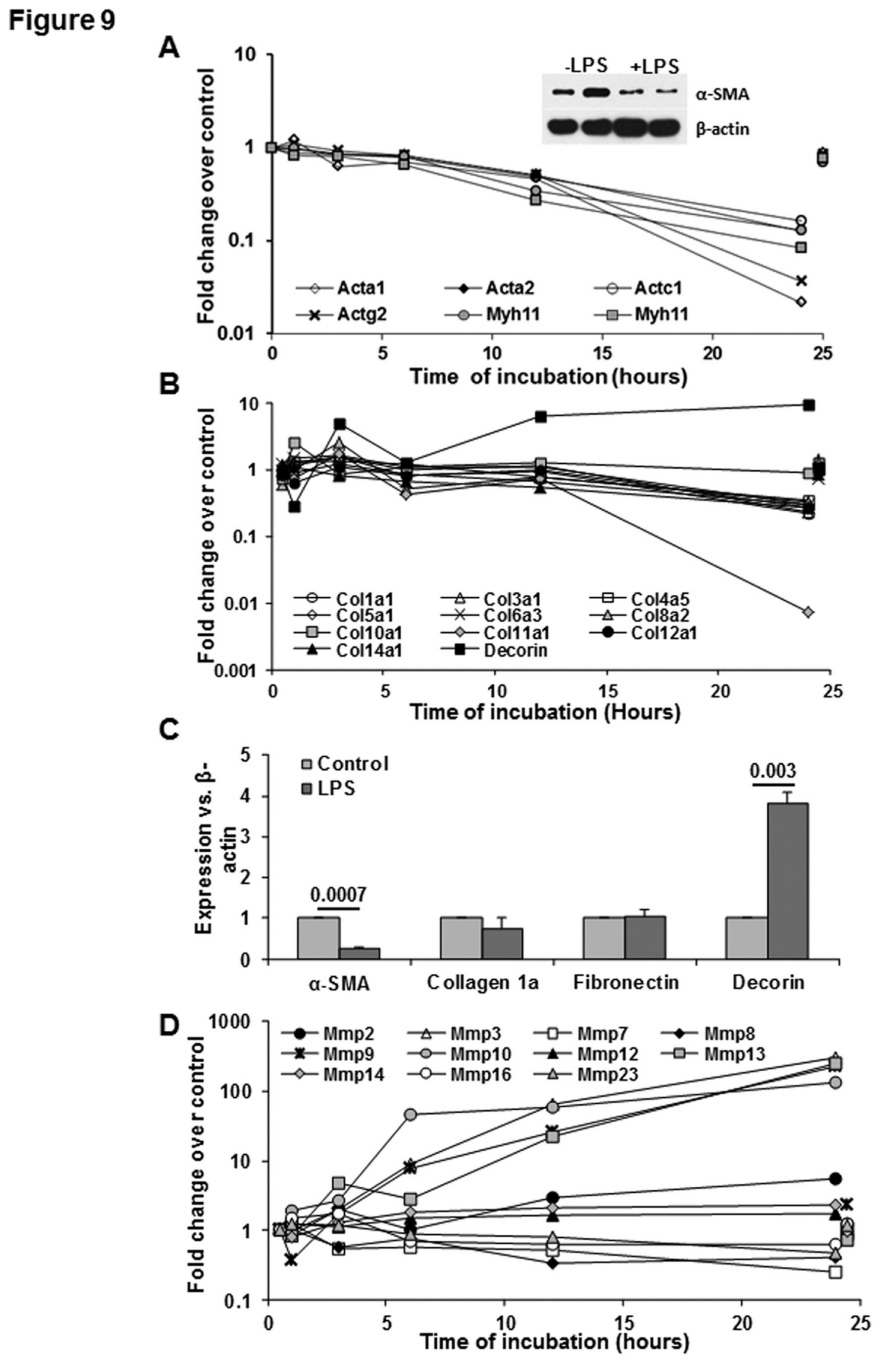
Effect of LPS on the genes associated with HSC activation. (A, B, D) Microarray data show LPS-induced time-dependent changes in the indicated transcripts. For clarity control gene expression at 1 and 24h is offset to 0.5h and 24.5h respectively. Inset in (A) shows a Western blot for α-sma protein expression in unstimulated and LPS (10 ng/ml)-stimulated HSCs. (C) qPCR data showing mRNA expression of the indicated molecules at 24h following stimulation with 10 ng/ml LPS. The numbers are p values form 3 separate determinations from different batches of HSCs. Statistical significance was derived from student’s *t*-test using Microsoft-excel program.

**Table 1:** Alterations in LPS-induced gene transcripts in HSCs.

	Earliest 2-fold change (h)	Max fold increase(Decrease in **bold**)	Time at max change (h)
LPS Response Elements (Figure 1)			
Cd14	1	14.0	24
Tlr2	3	9.0	6
Tlr7	1	16.8	6
Tlr8	6	**-2.9**	6
MAPK Signaling (Figures 2)			
Mapk14	6	**-2.7**	6
Mapk1	6	**-2.1**	6
Mapk6	12	3.5	12
Map2k1	12	2.6	12
Map2k3	24	3.1	24
Map3k1	6	**-3.5**	6
Map3k4	6	**-3.1**	6
Map3k8	1	18	6
Map4k4		(+70%)	24
NFkB Signaling (Figures 3)			
Nfkb1	3	7.1	6
Nfkb2	3	8.6	6
Nfkbia	1	23.3	1
Nfkbib	6	3.9	24
Nfkbiz	1	32.8	3
IRAK/STAT/SOCS Signaling (Figures 3)			
Irak2	3	6.9	6
Irak3	3	9.5	12
Stat1/Stat4	6	4.0	12
Stat2	3	9	12
Stat3	6	3	6
Socs3	3	6	3
Socs5	3	**-2.2**	3
Cytokines (Figures 4-6)			
Il1a	1	59.7	24
Il1b	1	137.9	12
Il6	3	79	12
Il10	12	242.7	24
Il17f	12	40.2	24
Il17ra		(**-48%**)	6
Il17re	6	**-5.2**	24
Tnf	1	419.5	3
Tnfaip6	3	44.8	24
Tgfb1		(+55%)	24
Tgfbr1	3	**-7.5**	24
Bambi	1	9.8	3
Irf1	3	15.2	3
Irf7	3	30	12
Irf9	3	3.7	12
C-C Chemokines (Figure 7)			
Ccl2	1	3.9	24
Ccl3	1	46.2	6
Ccl4	1	52.6	6
Ccl5	3	107.1	12
Ccl6	12	21.5	24
Ccl7	1	18.8	24
Ccl9	1	36.0	6
Ccl19	3	6.1	12
Ccl20	1	113.7	12
Ccl22	12	3.7	24
CXC Chemokines (Figure 7)			
Cxcl1	1	52.3	6
Cxcl2	1	316.8	24
Cxcl3	3	144.8	24
Cxcl5	3	23.2	6
Cxcl9	3	23.8	6
Cxcl11	1	146.2	6
Cxcl13	6	4.3	24
Cxcl16	6	5.0	24
Surface molecules of immune cell interactions (Figure 8)			
Cd40	3	186.0	6
Vcam1	1	180.8	3
Cd86	3	9.6	6
RT1-EC2	3	11.6	6
RT1-CE12	6	3.9	24
RT1-T24-3	3	11.7	12
RT1-A2 / RT1-A3 / RT1-EC2	6	4.2	24
RT1-M3-1	6	2.5	12
RT1-N3	6	2.9	6
RT1-S3	3	26.4	12
RT1-DMa	12	**-4.4**	24
RT1-DMb	12	**-4.5**	24
Cd200	6	11.0	12
Markers of stellate cell activation (Figure 9)			
Acta 1	12	**-45.0**	24
Acta2	24	**-7.6**	24
Actg2	24	**-25.9**	24
Decorin	1	10.9	24
Col1a1	24	**-4.4**	24
Col3a1	24	**-4.0**	24
Col1a1	24	**-4.4**	24
Col4a5	24	**-2.9**	24
Col5a1	24	**-3.9**	24
Col8a2	3	4.4	3
Col11a1	3	**-110.5**	24
Col12a1	24	**-4.0**	24
Col14a1	12	**-4.3**	24
Mmp2	3	5.6	24
Mmp3	6	308.1	24
Mmp7	24	**-4.1**	24
Mmp9	6	230.4	24
Mmp10	6	133.1	24
Mmp13	6	255.4	24
Mmp14	12	2.3	24
Mmp23	24	**-3.9**	24

### Expression of LPS response elements by HSCs

The liver is the major target of gut bacteria-derived pathogen-associated molecular patterns (PAMPs) such as LPS, peptidoglycans and RNA/DNA. PAMPs are recognized by the specific toll-like receptors (TLRs): lipoproteins by TLR1 and TLR2, double stranded (ds)RNA by TLR3, LPS by TLR4, Flagelin by TLR5, single stranded RNA by TLR7 and TLR8, and CpG-containing DNA by TLR9. The cell’s response to the PAMPs is dependent upon the presence and association of TLRs with their co-receptors and adaptor molecules (e.g., CD14, MD2 and MyD88) [31]. LPS elicited variable effects on the various response elements. LPS increased expression of Cd14 transcript but not of Myd88 and Md2 (Figure 1A), which was consistent with their protein expression (Figure 1B). Tlr1, Tlr2, Tlr4, Tlr7 and Tlr8 all were detected in HSCs (Figure 1A). LPS did not alter the expression of Tlr1orTlr4 appreciably, but increased Tlr2 and Tlr7, consistent with the qPCR analysis (Figure 1C). Western blot analysis also showed no alteration in TLR4 expression by LPS (Figure 1B). We previously observed very low mRNA expression of TLR4 in quiescent rat HSCs, while transitionally activated HSCs showed noticeable TLR4 expression [14]. Quiescent human HSCs were also reported to express low levels of TLR4 mRNA, which was up-regulated by activation in culture [32]. In contrast, mouse-derived HSCs were reported to express TLR4 at the same level in both quiescent and activated states [18], and activated human and mouse HSCs respond to LPS via TLR4 and not TLR2 [18,32]. Brun et al [33] reported that exposing activated murine HSCs to LPS, LTA or N-acetyl muramyl peptide caused phosphorylation of ERK1 and subsequent up-regulation and release of TGFβ1, IL6, and monocyte chemoattractant protein 1 (MCP1, encoded by Ccl2). In C3H/HeJ mice lacking TLR4, LPS-mediated effects were attenuated, but not blocked arguing for an additional TLR4-independent response to LPS. Indeed, we previously reported LPS responses in quiescent rat HSCs, which have minimal expression of TLR4 [17].

Tlr8 was down-regulated up to 12h with return to the basal level by 24h. The microarray did not survey Tlr9 but qPCR demonstrated its presence in HSCs, which was, interestingly, down-regulated by LPS (Figure 1C). The robust LPS-induced increase in the transcript for Tlr7 (Figure 1A, 1C) is interesting in that TLR7 is located in the membranes of the endosomal compartment and recognizes ssRNA, a common feature of viral genomes that are internalized by macrophages [34]. As TLR7 has been postulated to play a significant role in HCV-induced immune responses [35], our results suggest a direct role for HSCs in responding to viral infection. In this regard, HCV RNA has been shown to incorporate into HSCs resulting in decreased expression of collagen (COL)1A1, COL4A2, COL5A1 and COL15A1, and increased expression of MMP1, MMP3 and MMP12 [36]. However, another study observed significant correlation of TLR7 levels with the grade of necro-inflammation, and more advanced stages of liver fibrosis [37]. Thus, it will be important to ascertain the precise role of HSCs in hepatic immune response related to HCV RNA-TLR7 interactions. Together, these observations suggest differential responses of HSCs to LPS depending upon the species from which they are derived and the state of activation.

#### LPS-induced signaling coupled to cytokine and chemokine synthesis

The gene and protein expressions of the cytokines and chemokines as well as other biological mediators are regulated directly or via cross-talk between various intracellular signaling pathways such as NFkB, MAPKs (mitogen-activated protein kinases), IRAK (IL1 receptor associated kinase), STAT (signal transducer and activator of transcription) and SOCS (suppressor of cytokine signaling). 

#### MAPKs

Surveying 12 of the first tier (Mapk) genes and nine of their associated / interacting proteins, 7 and 8 respectively are expressed in at least six of the 11 samples. Transcripts of interest include Mapk3 (ERK1), Mapk1 (ERK2), Mapk6 (ERK3), Mapk9 (JNK2) and Mapk14 (p38) (Figure 2A). Of these components, Mapk14 shows a valid 2-fold decrease (6h), while Mapk1 expression is decreased to 50.6% of control at the same time-point. Mapk6 shows a valid increase at 12h, raising the possibility that increased ERK3 signaling may compensate for decreases in ERK2 and p38. The second tier (Map2k) transcripts surveyed are members 1 through 5, of which the first four are consistently present. Increased expression was seen for both Map2k1 and Map2k3 (which target ERK1/ERK2 and p38 respectively) while neither Map2k2 nor Map2k4 showed valid changes. If the higher transcript levels result in higher Map2k activities, these increased activities will give higher fractional phosphorylation of their respective (decreased) Mapk targets, repleting the respective Mapk activities. Of 11 third tier (Map3k) genes surveyed, 8 were substantially present, of which Map3k1 (encodes MEKK1) and Map3k4 (encodes MEKK4) showed decreased expression at 6h; MEKK1 activates both Erk1/2 and Jnk2. The strong up-regulation (max 16 -fold) of Map3k8 (Cot/Tpl2) is interesting as *Tpl2* ^*-/-*^ mice are resistant to endotoxic shock, and derived HSCs show decreased ERK-dependent upregulation of IL1β and Timp1 in response to LPS [38]. Cot/Tpl2 is activated by IL1, TNFα and LPS via the IκB kinase-beta (IκBKβ)-catalyzed phosphorylation of the p105 regulatory subunit [39]. Cot/Tpl2, which is essential for LPS-induced activation of the MEK/ERK and JNK kinase pathways, plays an integral role in the production of pro-inflammatory cytokines such as TNFα and IL1β in macrophages [40], and of TNFα and IL2 during T lymphocyte activation [41]. Cot/Tpl2 was also shown to activate IkB kinases, thus inducing the nuclear translocation of NFkB. We previously observed inhibition of LPS-induced H2O2, NO, TNFα, and IL6 synthesis upon blocking activation of p38-MAPK and NFkB, but not of ERK1/2- and JNK-MAPK [17]; since the blockade of p38 MAPK prevented LPS-induced NFkB activation, the microarray data suggest that Cot/Tpl2 might regulate NFkB activation in HSCs by inducing p38 phosphorylation initially. However, contrary to this supposition, Cot/Tpl2 deficiency was shown to increase LPS-induced activation of p38 and JNK MAPK and expression of iNOS, and inhibition of PI3K or mTor prevented these regulatory effects of Cot/Tpl2 [42]. Thus simultaneous activation of both Cot/Tpl2 and p38/NFkB in LPS-stimulated HSCs might indicate counter-regulatory mechanisms during inflammatory response. In this regard, anti-inflammatory IL10 which is produced by LPS-stimulated Kupffer cells has been found to down-regulate synthesis of TNFα and IL6 by the same cells [43].

All of the five genes in the fourth tier (Map4k) are present and stable, except Map4k4, which shows a 2-fold increase at 24h. Map4k encodes mitogen-activated protein kinase kinase kinase kinase 4, an enzyme that activates MAPK8/JNK. In intact mice, siRNA silencing of Map4k4 expression protects against LPS lethality by inhibiting production of TNFα and IL1β [44]. Since LPS-stimulated HSCs produce these mediators, their contribution to the liver injury via MAP4K4 signaling may be significant. 

#### NFkB

The activation and subsequent loss of NFκB protein from the cytosol [17] is accompanied by substantial increases in the transcripts for NFκB p105 (Nfkb1), NFκB p49/100 (Nfkb2) and IkBα (Nfkbia), and 3.6-fold for IkBβ (Nfkbib) (Figure 2B). These changes replenish this key signaling system. While Nfkbia increases most rapidly (at 1h), the other 3 components of the system lag somewhat, increasing between 1 and 3h. Interestingly, the novel zeta (ζ) form of IkB (Nfkbiz) increases as rapidly as Nfkbia. IkBζ, which is hardly detectable in resting cells, is strongly induced by LPS or IL1β, but not by TNFα; localizing in the nucleus, it inhibits NFkB activity [45]. The microarray data thus suggest that the increased IkBζ expression may be a negative regulator of NFκB-induced pro-inflammatory and/or other (e.g., activation-related and pro-fibrogenic) effects of LPS. 

#### IRAK, SOCS and STAT

The microarray results also indicate that LPS promotes inflammatory response in HSCs by instigating cross-talk between IRAK, TLR and NFkB signaling. While the Irak1 transcript was slightly decreased throughout the time-course of LPS stimulation of HSCs, Irak2 and Irak3 transcripts increased strongly by 6h (Figure 3A), which was confirmed by qPCR (Figure 3B). IRAK2 is involved in the MyD88-dependent response that occurs on dimerization of the TLR receptor, and is utilized by every TLR except TLR3. MyD88 recruits IRAK1 and IRAK2, and IRAK kinases phosphorylate and activate the protein TRAF6, which in turn polyubiquitinates the protein TGFβ-activated kinase (TAK1) as well as itself in order to facilitate binding to IKKβ. On binding, TAK1 phosphorylates IKKβ, which then phosphorylates IκB causing its degradation and allowing nuclear translocation of NFκB to activate transcription and consequent induction of inflammatory cytokines [46].

 Stat1/Stat4 and Stat2 expression increased gradually by 12h, and declined somewhat at 24h. In contrast; increase in Stat3 expression was stable till 24h (Figure 3C). While Socs3 increased rapidly and remained stable, Socs5 expression declined during LPS stimulation up to 6h and then returned to the basal level (Figure 3C) confirmed by qPCR in Figure 3D. LPS-induced changes in STAT/SOCS expression can be important in the fibrogenic activity of HSCs. For example, IL22-induced activation of STAT3 and SOCS3 has been implicated in promoting senescence of HSCs thereby ameliorating fibrosis [47]. In another study, however, adiponectin-induced SOCS3 activation was found to prevent leptin-mediated fibrogenic activity of HSCs by inhibiting STAT3 signaling [48]. IFNγ has been shown to induce quiescence in pancreatic stellate cells via activation of STAT1 [49]. Clearly, these varied responses of LPS-induced signaling molecules (IRAK, STAT and SOCS) indicate instigation of stimulatory and counter-regulatory pathways in hepatic inflammation and immunity.

 The expression of other genes that control cell growth, proliferation and survival such as Mtor that encodes mammalian target of rapamycin (mTOR), and related genes Rptor, Lamtor1, Lamtor2 and Mlst8 were all present in HSCs and unaffected by LPS stimulation, while Rictor was absent throughout. Cot/Tpl2 (Map3k8), which controls Akt/mTor/p70S6k signaling, is increased in LPS-stimulated HSCs (Figure 2A). LPS has also been shown to stimulate the synthesis of IL10 and IL6 in PBMCs [50], and of TNFα and IL10 in peritoneal macrophages via mTor activation. However, inhibition of mTor reduces IL10 and increases TNFα synthesis [51] indicating mTor signaling is primarily anti-inflammatory. In this regard, inhibition of mTor augments LPS-induced lung injury [52] and its stimulation by resveretrol in microglial cells inhibits inflammatory response by down-regulating NFkB and MAPK signaling [53]. These data suggest that the interactions between LPS-induced mTor and other signaling pathways in HSCs as potential regulators of hepatic inflammation and immune responses. 

### LPS-induced cytokine expression

#### Interleukins, TNF family and nitric oxide

Microarray findings showed robust increases in Il6, Il1β, Il1α, Tnfα and Nos2 (encodes iNOS) in LPS-stimulated HSCs (Figures 4A-C). The data agree with our previous observation that while the Il6 transcript was still robustly elevated at 24h, the Tnfα transcript peaked at 3h and decreased thereafter [17]. The validity of the microarray data was confirmed by increased release of TNFα, IL6, IL1α and NO metabolites (NO2+NO3) by LPS-stimulated HSCs (Figure 4D). The LPS-induced increase in TNFα was also validated by intracellular staining of the cells and determination via flow (Figure S4). Moreover, changes in each of these components were accompanied by changes in family members or in functionally related genes (Figures 4A-C). For example, Il6, Il1α and Il1β up-regulation was accompanied by a modest decrease in gene expression of the respective receptors IL6ra and IL1rl1 (Figure 4A). On the other hand, gene expression of another receptor for IL1 family of cytokines, Il1r2, was unchanged. Also, up-regulation of iNOS (Nos2) was accompanied by >20-fold down-regulation of both heterodimer components (Gucy1a3 and Gucy1b3) of the soluble guanylate cyclase, which comprises the intracellular receptor for NO, presumably to attenuate the autacoid effects of elevated NO on intracellular signaling (Figure 4C). 

Il1α and Il1β transcripts showed early high increase (0-1h), Il6 increased early (maximum rate at 1-3h), and Il10 showed a slow, nearly linear increase from 0 to 12h. While culture activation of HSCs is known to cause an increase in IL10 mRNA, additional stimulation with LPS, TNFα or TGFβ further increases IL10 mRNA by 2-fold, resulting in greater release of IL10 protein into the medium [19,54]. Thus the basal as well as LPS-stimulated HSC-derived IL10 is a significant source of anti-inflammatory and pro-tolerogenic environment in the liver. 

Expression of Il1rn, which encodes the Ilr1 receptor antagonist, increased >100-fold on LPS treatment. This will likely inhibit the inflammatory signaling of IL1α and IL1β, and its downstream effects. LPS also increased gene transcript of a novel secreted cytokine IL17F by more than 10-fold by 24h stimulation, but did not affect the other members of IL17 family, IL17b and IL17d. Although Il17a is not surveyed by the microarray, IL17A protein was not detected in the culture supernatants of control or LPS-stimulated HSCs [19,20]. IL17F bears homology to IL17A, and is expressed and released by activated CD4^+^ memory T cells, the CD45-RO^+^ subset of CD8^+^ memory T cells and activated monocytes [55]. Recently, IL17 was reported to promote liver fibrosis in mice by activating inflammatory cells and KCs, and to stimulate collagen synthesis in HSCs directly through activation of STAT3 [56]. Since IL17A and IL17F share the same receptors (IL17Ra and IL17Rc) [55], a similar fibrogenic effect of IL17F via autocrine pathway in HSCs cannot be ruled out. However, microarray analysis showed nearly 2-fold (to 52% of control) LPS-induced decrease in Il17ra expression at 6h, and Il17rc was absent from all samples; the transcript of another receptor for IL17, Il17re was also robustly decreased from 6h onwards (not shown). These data indicate that such down-regulation by LPS may be a mechanism of limiting fibrogenesis during chronic liver injury. This effect is also relevant to LPS-induced down-regulation of the molecules coupled to HSC activation as shown later (Figures 5 and 9). The other important effects of IL17F are its potential to inhibit angiogenesis and to stimulate IL2, TGFβ, and MCP1 synthesis by human endothelial cells, although it does not stimulate the proliferation of hematopoietic progenitors or the migration of mature leukocytes [57]. Thus, expression Il17f by HSCs and its increase by LPS demonstrate a unique regulatory potential of these cells in hepatic angiogenesis and inflammatory/immune responses.

Consolidation of data from our previous reports on mouse HSCs [19,20], the present ELISA (Figure 4D) and microarray findings (see GEO data) show that rat HSCs do not express the cytokines typically associated with T cell differentiation/proliferation (IL2, IL4, IL5, IL13 and IL12p70), without or with LPS stimulation. Of interest is the observation that isolated KCs produce IL13, an anti-inflammatory protein [58] and not IL10 under basal conditions [59], and LPS stimulates secretion of IL10 [43,59] but not of IL13 in these cells [59]. This property of KCs is quite distinct from the significant spontaneous production of IL10 by HSCs and its stimulation by LPS (Figure 4) [19]. 

 Among the TNF-related genes, other than Tnfα, Tnfaip6 (encodes TNFα-Induced Protein 6: TNFAIP6) and Tnfrsf5 (encodes type I transmembrane glycoprotein CD40) showed nearly 100-fold increase by 3h of LPS stimulation that remained stable for 24h Tnfsf9 (encodes transmembrane cytokine CD137L) showed a transient increase at 1h after LPS stimulation, and returned to the basal level by 6h (Figure 4B). The expression of Tnfaip6 by HSCs and its up-regulation by LPS suggests a critical regulatory mechanism in liver pathophysiology considering the involvement of TNFAIP6 in stabilizing extracellular matrix and cell migration through a hyaluronan-binding domain, and also in protease network associated with inflammation by forming a stable complex with inter-α-inhibitor and enhancing its serine protease inhibitory activity. 

#### TGFβ and related genes

LPS induced down-regulation of Tgfb2 and Tgfb3 in HSCs (Figure 5A). Tgfb1 transcript was down-regulated by 1.4-fold at 1-3h and up-regulated by 1.5-fold at 24h. However, qPCR analysis showed decrease in TGFβ1 mRNA at 24h (Figure 5B). In our experience, quiescent and transitionally activated HSCs are not major sources of TGFβ1. Interestingly, LPS also strongly down-regulated Tgfbr1 transcript throughout the time-course, and up-regulated the transcript for TGFβ pseudoreceptor BAMBI (BMP and the activin membrane bound inhibitor) up to 3h that decreased to the basal value thereafter (Figures 5A, 5B). These data with transitionally activated rat HSCs differ from that with quiescent mouse HSCs in which LPS did not affect Tgfbr1 expression but down-regulated BAMBI [18]. It was proposed that by down-regulating BAMBI, LPS enables HSCs to react with macrophage-derived TGFβ during hepatic fibrogenesis. Our data, however, suggest that LPS may exert counter-regulatory actions by up-regulating BAMBI and down-regulating Tgfbr1 expression following the initial period of liver injury when HSCs are undergoing activation. It is likely that these variable results may also reflect species-specific effects.

### LPS-induced interferon expression and downstream effects

The microarray surveys Interferons (IFN) α1, β1, γ and κ, also receptors Ifnar1, Ifngr1 and Ifngr2. Of these, IFNs γ and κ were undetectable in any sample, while transcripts for Ifnα1 and for receptors were detectable and unchanged. In contrast, Ifnβ1 showed a very strong transient increase. (see GEO dataset). To assess the potential autacoid effect of the IFNβ1 transient we assembled a list of known IFN-modulated genes using the NCBI Gene database, the Interferome.org database, and supplementary data from Schoggins et al. [60]. From the Interferome.org database we used only genes which had been cited in more than one literature report. The aggregate list contained 372 genes surveyed by the microarray. Of these 207 (55%) were modulated in at least one experiment with increased expression of 76, 1, and 81 genes respectively in experiment 1 only, experiment 2 only, and concordantly. Decreased expression occurred for 21, 6, and 15 genes respectively, so that increases represent 158 of the total 200 genes (79%). The remaining seven genes were modulated discordantly. Contingency analysis (χ^2^ test) showed that twice as many IFN-responsive genes were modulated as would be expected by random chance, (-log(p) = 28.8 ) confirming a robust response to autacoid IFNβ1. We note that five of the ten Cxcl and five of the ten Ccl family members increased (see below) are IFN-inducible.

Of nine interferon regulatory factors (Irfs) all are surveyed except -4 and -8; Irf6 was essentially absent and only -1, -7 and -9 showed robust increases (Figure 6A). IRF1 is reported to be a major mediator of ischemia/reperfusion injury that occurs upon partial liver resection and transplantation of the cold/ischemic preserved graft [61,62]. Expression of IRF1 in inflammatory cells (e.g., DCs) is coupled to the production of type 1 IFNs (IFNα and IFNβ); the released type 1 IFNs then induce IRF1 expression in hepatocytes, which initiates hepatocyte death via HMGB1 [63]. It is likely that a similar mechanism of hepatocyte injury may be mediated by LPS-stimulated HSCs, with IRF1 as an inducer of inflammatory mediators in HSCs. In contrast, the gene expression of IRF2, an antagonist of IRF1, did not change in response to LPS, suggesting that the IRF1 pathway is primarily skewed toward hepatocyte injury. While the Irf1 transcript decreased somewhat following initial up-regulation, Irf7 transcript increased in a stable manner still showing maximal (about 50-fold) increase at 24h. qPCR results show very weak expression of IRF7 that increased 150-fold upon LPS stimulation (Figure 6B). IRF7, whose expression has been shown to be restricted to certain cells types such as B cells and DCs [64], is a key innate immune molecule in the type I IFN signaling pathway, and is essential for the type I IFN response to many viruses [65]. It is also known that IRF7 forms a complex with MyD88, which causes activation of IFNα-dependent promoters [66]. IRF9 is a crucial factor for accelerating IFNα-induced early antiviral signaling [67]. These data show that HSCs instigate a robust type I IFN response through IRF1- and IRF7-induced transcriptional activity, with IRF9 optimizing the timing and size of the response.

Taken together, these data show that LPS stimulates a transient IFNβ1 response, with subsequent autacoid modulation of a very substantial population of IFN-sensitive genes. 

#### LPS effects on CC (cysteine-cysteine) chemokines and their receptors

In the CC chemokines, Ccl3 (encodes MIP1α), Ccl4 (encodes macrophage inflammatory protein-1β: MIP1β), Ccl5 (encodes RANTES: regulated on activation, normal T cell expressed and presumably secreted), Ccl6, Ccl7, Ccl9 (encodes macrophage inflammatory protein-1 gamma: MIP1γ), Ccl19 and Ccl20 (encodes macrophage inflammatory protein 3α [MIP3α] or Exodus-1, also known as liver and activation-regulated chemokine [LARC]) all show >10-fold increases, while Ccl2 (encodes MCP1) and Ccl22 (encodes macrophage-derived chemokine [MDC]) show about 4-fold increases (Figures 7A, 7C). We have previously shown LPS-induced synthesis of CCL2 and CCL3 [19], and thus the microarray findings are consistent with LPS-induced ERK1/2 phosphorylation [17,33], which is coupled to up-regulation of CCL2 and CCL3 [33,68]. The proteins encoded by these chemokine genes display strong chemotactic activity towards various inflammatory and immune cells (Table 2). The increase in CCL2-5 expression in KCs and endothelial cells was proposed to be an important determinant in a murine model of fulminant hepatic failure (FHF) and in human FHF patients due to migration of CD14-positive macrophage and CD3-positive lymphocytes [69]. Our data suggest that HSCs may also contribute significantly to this pathology. On the other hand, CCL2 and CCL3 also induce migration of DCs, and their subsequent interaction with HSCs renders them tolerogenic suggesting their protective role in liver allograft transplantation [20].

**Table 2:** Chemokines expressed by HSCs and their target cells.

**Chemokine**	**Target cells**
CCL2	Monocytes/macrophages, DCs and memory T cells
CCL3	Monocytes, NK cells and memory T cells
CCL4	NK cells, NKT cells, monocytes, Tregs, B cells and DCs
CCL5	Eosinophils, basophils, NK cells and T cells
CCL7	DCs, Monocytes and macrophages
CCL9/CCL10	CD34+ immature myeloid cells and DCs
CCL19	DCs, B cells, memory T cells and Tregs
CCL20	T cells and neutrophils
CCL22	Monocytes, DCs, NK cells, activated T cells and Tregs
CXCL1	Neutrophils, monocytes and T cells
CXCL2	Neutrophils, eosinophils, basophils
CXCL3	Eosinophils, basophils, monocytes, DCs and T cells
CXCL4	Neutrophils, eosinophils, basophils
CXCL5	Neutrophils, eosinophils, basophils
CXCL9	NKT cells, CD4 T cells, CD8 T cells
CXCL10	T cells, neutrophils, monocytes. NK cells
CXCL11	NKT cells, CD4 T cells, CD8 T cells
CXCL13	B cells
CXCL16	NKT cells, CD4 T cells, CD8 T cells

DCs, dendritic cells; NK, natural killer cells; Tregs, regulatory T cells.

Highly activated human (passage 3-9) HSCs were found to express CCL5 and its receptor CCR5 (70). NFkB-activation by TNFα, IL1β and CD40L increased CCL5 expression, which caused migration and proliferation of HSCs [70]. The present microarray data with transitionally activated rat HSCs also demonstrate expression of CCL5, which increases upon LPS stimulation, and is consistent with increased LPS-induced release of the chemokine in the medium (Figure 7A; inset). Higher CCL5 gene expression levels were observed in activated murine HSCs after stimulation with TLR3 ligand poly I:C as compared to that with LPS [71]. Since CCL5 mRNA levels are suppressed during early fibrosis by Peg-IFNα in the HCV infected patients, it was proposed that poly I:C-induced CCL5 gene expression in HSCs may be involved in regulating fibrogenesis [71]. Our data showing robust increase in CCR5 expression (about 95-fold by 6h) in LPS-stimulated HSCs (results not shown) indicate that autocrine CCL5/CCR5 interaction may be a critical event contributing to the liver damage during acute liver injury and then fibrogenesis. We note that the chemokines released by LPS-stimulated HSCs were also shown to play an important role in biliary obstruction-induced hepatic fibrosis in a mouse model [18]. 

Among the other receptors of this class, CCR2 (CCL2 and CCL7), CCR3 (CCL7), CCR4 (CCL7 and CCL22), CCR6 (CCL7, CCL20) and CCR10 (CCL7) were undetectable by microarray in HSCs without or with LPS stimulation, CCR7 (receptor for CCL19), CCR8 and CCR9 are not addressed by the microarray, and CCR1 was expressed by HSCs and its expression increased by 300-fold upon LPS stimulation (results not shown). The latter results suggest that autocrine effects of CCL3, CCL6 and CCL9 on HSCs via their receptor CCR1 can be of potential importance in hepatic pathophysiology. Interestingly, CCR2, which is mainly expressed by monocytes and macrophages [72-74], was also reported to be expressed by mouse HSCs, and based on reduced bile duct ligation- and CCl4-induced fibrosis in CCR2^-/-^ mice, it was concluded that CCR2 is responsible for migration of HSCs during liver injury [72]. 

#### LPS effects on CXC (cysteine-X-cysteine) chemokines and their receptors

In the CXC chemokines, Cxcl1 (~50-foldmax), Cxcl2 (~100-foldmax), Cxcl10 (~100-foldmax) and Cxcl11 (~100-foldmax) increased very rapidly at 1-3h, Cxcl3, Cxcl5 and Cxcl9 increased with a slight delay with maximal increase of ~50-100-fold at 3-6h, while Cxcl13 and Cxcl16 increased gradually by about 5-fold by 6h of LPS stimulation (Figure 7B, 7C). Expression levels of all of these genes remained stably elevated up to 24h, except that of Cxcl10 that decreased to ~20-fold by 24h. Cxcl4 and Cxcl12 did not show any appreciable change in response to LPS. The robust and rapid increase in Cxcl1, Cxcl2, Cxcl4 and Cxcl5 indicate the strong potential of HSCs in inflammation, angiogenesis (e.g., during liver regeneration) and wound healing by causing chemotaxis of neutrophils, eosinophils and basophils. Interactions of these pathways with IL17F, which is up-regulated by LPS (Figure 4A), can be critical in angiogenic role of HSCs. LPS-induced up-regulation of Ccl2, Cxcl1 and Ccl3 in HSCs, which suggests their involvement in the recruitment of monocytes and neutrophils, is supported by our recent observation showing decreased LPS-induced hepatic infiltration of neutrophils in HSC-depleted mice [30]. HSCs may also play a direct role in innate immunity since CXCL1 and CCL3 (MIP1α) possess anti-bacterial and anti-viral activity, respectively. Furthermore LPS-induced up-regulation of Cxcl10 (encodes IP10) (Figure 7B) and secretion of IP10 by HSCs [19,20] provide an additional mechanism of HSC-mediated recruitment of lymphocytes, neutrophils and monocytes in the liver. 

In addition to Cxcl10, LPS also up-regulated the expression of Cxcl9 (encodes MIG), Cxcl11 and Cxcl16 indicating that HSCs can induce migration of NKT cells and conventional CD4 and CD8 T cells. The ability of HSCs to present bacterial lipid antigens to NKT cells has been shown to be a significant factor in the control of bacterial infection [24]. Furthermore, HSCs suppress proliferation and induce apoptosis of conventional T cells directly as well as by modulating DCs to a tolerogenic phenotype and by expanding Tregs that acquire superior immunosuppressive potential [19,20,25]. Our data also shed light on the possible contribution of HSCs in a murine model of FHF in which administration of heat-killed *Propionibacterium acnes* followed by a low dose of LPS increased the expression of Ccl5, Cxcl9, Cxcl10, Cxcl16 (the ligand of Bonzo, CXCR6) and TNFα [75]. Of note is the observation that up-regulation of Cxcl16 closely correlated with the magnitude of immunological liver injury induced by BCG-LPS in mice [76], and intrahepatic recruitment of specific lymphocytes by HSCs might be an important mechanism of this injury. Moreover, as described above, LPS-up-regulated Ccl2-5 in HSCs can also contribute to liver failure. 

Among the Cxcr class of receptors, rat HSCs did not express Cxcr1/Il8ra, Cxcr2/IL8rb and Cxcr5/Blr1, and Cxcr6 is not surveyed by the microarray. Some differences appear in the rat and mouse HSCs in regard to the expression of CXCL1 and CXCR2. While activated but not quiescent mouse HSCs expressed CXCL1, both phenotypes expressed its receptor CXCR2 [77]. However, CXCR2 was expressed by the liver after CCl4-induced injury followed by administration of CXCL1, leading to a concluded that CXCL1/CXCR2 interaction plays an important role in liver fibrosis [77]. Interestingly, HSCs express Cxcr3, Cxcr4 and Cxcr7 transcripts, and LPS modulated the last two; Cxcr4 is predominantly down-regulated, while modulation of Cxcr7 is complex (see GEO data). CCR5 and CXCR4 are the two major co-receptors required for HIV entry into cells, and the primary HIV isolates were found to infect both human HSC line, LX-2, and primary human HSCs via CCR5 and CXCR4, and to promote collagen I expression and secretion MCP1 [78]. Together, these data provide strong additional evidence for HSCs to modulate hepatic microenvironment by attracting and then interacting with inflammatory and immune cells that have potential implications in innate and adaptive immunobiology.

#### Antigen-presenting and co-regulatory molecules

Several reports have shown HSC’s ability to influence T cells by presenting Ag in conjunction with co-modulatory molecules (e.g., CD80, CD86, B7-H1). For example, bacterial lipid Ag presentation by HSCs activates NKT cells and regulates microbial infection [24]. On the other hand, HSCs induce expansion of Tregs via MHC II [19], cause apoptosis of T cells via B7H1 (PD-L1) [25], suppress CD8 T cell activation in a CD54-dependent manner [26], and inhibit CD8 T cell activation/proliferation via B7H4 [27]. Previously, we found that LPS induces strong increase in MHC class II and co-stimulatory molecules CD80 and CD86 as well as VCAM, and smaller increase in MHC class I, CD40, CD54 and B7H1 in mouse HSCs [19]. In the present study with rat HSCs, the B7H1 homolog (CD274/Pdcd1lg1) is not surveyed by the microarray (see Figure 8A), and Cd80 transcript was undetectable in any sample. However, Cd86 and Vcam1 transcripts increased in LPS-stimulated HSCs concordantly with protein expression (Figure 8A). Additionally, the increase in the transcript for Cd40 was robust, while modest increases were observed in the transcripts for Icam1 and specific MHC I molecules. Cd40 and Icam1 transcripts peaked at 6h of LPS treatment, and by 24h had fallen by 80% and 44% respectively. This likely explains the previously observed low increases in the respective encoded proteins in mouse HSCs at 24h of LPS stimulation [19]. Alternatively, mouse HSCs may respond differently to LPS as discussed above for TGFβ related molecules, and chemokines.

Cells of myeloid origin including DCs express receptor for a glycoprotein OX2 or CD200; CD200R is an inhibitory receptor that affects myeloid cell functions [79-81]. We have observed CD200R in liver mDCs and surface expression of CD200 on HSCs, which is increased by LPS (not shown). These data are consistent with expression of Cd200 transcript and its 10-foid increase by LPS within 6h (Figure 8B). We are currently investigating CD200/CD200R interactions as an important the mechanism of our prior finding that HSC-modulated DCs are poor stimulators of T cells [20]. 

### LPS down-regulates activation state of HSCs

Treatment of culture activated rat or human HSCs with recombinant human IFNβ (rhIFNβ) causes decreased expression of α-SMA, collagens I and III, TGFβ1, PDGF-BB and Smad4, while Smad7 expression is increased [82]. The present data show that LPS causes a robust autacoid IFNβ response (see above), so we reviewed concurrent cytoskeletal and fibrotic changes. PDGF-BB is not surveyed by the microarray, and we found Smad4 expression to be stable but Smad6 to decrease (see GEO dataset).

#### Cytoskeletal changes

A search of the root “cytoskel” in the NLM Gene database gave 1537 responses, of which 1252 are surveyed. Of these, 106 showed at least one valid increase and 218 showed at least one valid decrease. Acta2 encoding α-SMA, as well as both Acta1 and Actg2 (encoding skeletal muscle and smooth enteric muscle actins, respectively) and Actn1 (nonmuscle α-actinin 1) were robustly decreased. Nonmuscle myosin II (NMM II) is an actin-binding complex comprising myosin heavy chains II-A II-B and II-C encoded by Myh9, Myh10 and Myh14 respectively; regulatory light chains encoded by Myl12a, Myl12b and Myl9; and essential light chains such as Myl6 [83]. Myosin heavy chains (Myh) surveyed are 1, 6, 7, 8, 9, 10, 11, 13, 14, of which only 9, 10, 11 and 14 are detectable. Expression of Myh9 and Myh10 is decreased as is Myh11, which encodes a smooth muscle chain. Of eight Myl members surveyed (-1, -2, -3, -4, -6, -7, -9, -12b) -3 and -7 were undetectable and Myl9 was decreased; the remainder were stably expressed, with Myl6 showing the highest level. Of three Mylk (myosin light chain kinase) members, the detectable smooth muscle isoform (Mylk) was decreased by LPS while Mylk2 and Mylk3 (encoding skeletal and cardiac isoforms respectively) were undetectable. This confirms the robust expression of the nonmuscle myosin II complex in HSCs, and a decrease in four of seven detectable transcripts on stimulation with LPS. Other structural muscle components, (Figures 9A, 9C), including Cnn1, Cryab, Tagln and Tpm1 (see GEO data) were also down-regulated >5 fold. Nonstructural genes associated with muscle development or function including Dmpk, Dysf, Hspb6, Kcnmb1 and Csf1r were also down-regulated. 

#### Fibrogenic changes

Abnormalities in liver function, including chronic liver diseases, increase circulating LPS levels. Liver injury is associated with activation of HSCs and fibrosis suggesting that LPS might be involved in HSC activation. The present data show that while the mRNA encoding TGFβ1,TGFβ2 and TGFβ3 are either unaffected or decreased by LPS, the receptors Tgfbr1 and Tgfbr2 are decreased, and BAMBI is transiently up-regulated (Figure 5). Further examination revealed down-regulation of the genes associated with HSC activation such as Acta 2 (Figure 9A, 9C) as well as decreased expression of α-SMA, a protein encoded by this gene in LPS-stimulated transitionally activated rat HSCs (Figure 9A: inset). In contrast to this effect, LPS up-regulated Pdgfra transcript by about 10-fold in HSCs (see GEO dataset). The activation of HSCs is associated with increased expression of PDGF receptor that is a powerful stimulus for their proliferation by PDGF produced in the injured liver. Whether LPS signaling promotes, inhibits or has no effect on PDGF-induced proliferation of HSCs remains to be determined.

LPS decreased the microarray-assayed expression of several collagen transcripts by 24h, including Col1a1, Col3a1, Col4a5, Col5a1 Col8a1, Col12a1 and Col14a1 (Figure 9B). Col8a2 and Col11a1 showed early transient increases before decreasing at 24h. On the other hand, fibronectin expression was high and unchanged (see GEO dataset), while decorin expression was up-regulated by 10-fold at 24h of LPS stimulation. Confirmatory qPCR at 24h (Figure 9C) showed a trend to decrease in Col1a1, stable fibronectin expression, and increased decorin expression. Decorin has been shown to regulate collagen fibrillogenesis, to block bioactivity of TGFβ1 and to exert protective effect against fibrosis. Together, these data indicate that LPS exerts strong anti-fibrogenic effect in the liver by limiting or down-regulating activation-associated changes in HSCs. It will be of interest to determine whether LPS has similar effects on fully activated (passage 3-4) HSCs and those isolated from chronically injured fibrotic liver (e.g., after chronic 12-14 weeks of CCl4 treatment).

 HSCs express several matrix metalloproteinases (MMPs) (the zinc- and calcium-dependent proteases) that degrade ECM and other extracellular proteins, and their inhibitors (tissue inhibitors of MMP: TIMPs). These enzymes regulate ECM components in physiology and an imbalance in their relative expressions and activities during liver injury is responsible for ECM remodeling, repair during organ injury and fibrosis. The microarray data show strong (about 100-fold) up-regulation of the Mmp3, Mmp9, Mmp10 and Mmp13 transcripts, while Mmp12, -16, -17, -19 and -24 showed no change (Figure 9D). Timp1 transcript showed 3-fold increase while Timp2 transcript decreased slowly that was significant at 24h; Timp3 and Timp4 expression levels were not prominent and did not change over the time-course of LPS stimulation (see GEO database). Since MMP13 deficiency was shown to resist liver fibrogenesis due to biliary obstruction [84], and activated MMP13 converts pro-MMP9 to its active form MMP9, which induces HSC activation/transdifferentiation [85], these data suggest that this pathway favors fibrogenesis. Furthermore, decrease in Timp2 (an inhibitor of MMP13 activation) expression also favors the role for LPS in promoting fibrogenic activity of HSCs. These data are paradoxical to the down-regulation of HSC activation markers (Figure 9A) and up-regulation of MMP3 (degrades collagen types II, III, IV, IX, and X, proteoglycans, fibronectin, laminin, and elastin), which indicate that LPS also instigates mechanisms that ameliorate fibrogenic response of HSCs.

In summary, the functionally validated microarray data discussed here indicate a crucial role of HSCs in hepatic inflammation and immune regulation. Limitations on space preclude full discussion of other facets of the HSC response, e.g., modulation of growth factor expression (upregulation of Ngf, Hgf and Vegfa, downregulation of Hbegf, Igf1 and Vegfc), growth factor receptors (Egfr and Ogfr upregulated; Fgfr3 down-regulated) and up-regulation of the vasoconstrictor cytokine endothelin-1, all of which will affect the interaction of HSCs with nonimmune hepatic cells. Obviously, there are differences in some of the highly important LPS effects on mouse versus rat HSCs, and it cannot be ruled out that such differences may likely be observed in the responses of human HSCs to LPS. It should be considered that while the experimental rodents are maintained in controlled pathogen-free environment, humans are subjected to variable environmental and food-derived factors on a continuous basis, which can likely alter the responses of HSCs to LPS. Nevertheless, the present data are of potential importance in future investigations to discover the varied roles of HSCs in hepatic inflammation and immunity.

## Supporting Information

Figure S1 **Purity of HSCs.** (**A**) Unstimulated or LPS-stimulated HSCs were harvested using trypsin/EDTA solution, then stained with anti-CD31 (endothelial cell marker), anti-CD68 (Kupffer cell marker), anti-CD11b/c (myeloid cell marker) or anti-GFAP (HSC marker) Abs and subjected to FACS analysis on a LSR II Flow Cytometer. Upper panel shows the purity of HSCs (gated on total live cells), while lower panel shows the respective isotype controls. (**B**) Protein lysates of unstimulated (CT) or LPS-stimulated (LPS) HSCs were subjected to SDS-PAGE. Separated proteins were transferred on to PVDF membrane and immunoblotted with anti-CD11b , -CD31 or -CD68 Abs. After washing, the membranes were incubated with secondary Ab, and signals were detected using ECL Western blotting detection reagent (GE Healthcare/Amersham, Buckinghamshire, UK). Liver lysates were used for positive control. (**C**) HSCs on glass coverslips were fixed (with 2% paraformaldehyde), permeabilized and stained for α-SMA (red) and nuclear stain (DAPI). All the cells are stained positive for α-SMA and also contained vitamin A (green autofluorescence).   
(TIF)

Figure S2 **Pairwise comparisons of control vs LPS-stimulated stellate cells.** A contingency analysis of the agreement between the first and second experiments shows that genes with ≥3 of 10 valid 2-fold changes in the first experiment are more than twice as likely than chance would predict to be modulated in the second experiment. This concordance is so marked that for ≥ 7 changes in the first experiment there are ≥ 9-fold more genes with ≥ 3 changes than expected in the second experiment, and ≥2-fold fewer genes than expected with zero changes. This is a way of including concordance between genes which are modulated in the second experiment (where 1h and 24h are examined) with genes which respond in the first experiment at intermediate times (3h, 6h or 12h). Exceptions in the Graph: there is no enrichment for 5 changes in experiment 1 and 3 changes in experiment 2; and there are no genes which show 9 changes in experiment 1 and 2 changes in experiment 2 (gap indicated by grey doubled-headed arrow).
(TIF)

Figure S3 **Pairwise comparisons of all control vs stimulated comparisons within each experiment.** For experiment 1 this yields (2 control x 5 stimulated = 10 comparisons), while experiment 2 yields (2 control x 2 stimulated = 4 comparisons), for a total of 14 pairwise comparisons. The 10,903 unique characterized genes which shown (14 ≥ n ≥ 0) valid 2-fold differences are shown (unfilled bars). 1,692 unique characterized genes were found with values distributed so that p < 0.042 by the Mann-Whitney test. The numbers of these genes which have corresponding two-fold changes also are shown (filled bars); the fraction they represent of those corresponding changes is shown (filled circles, right hand axis). The progressive increase of this fraction is consistent with a predominant directionality in the modulated genes; i.e. most genes are consistently upregulated or downregulated.
(TIF)

Figure S4 **Intracellular staining of TNFα in stellate cells.** Unstimulated or LPS-stimulated HSCs were harvested using trypsin/EDTA solution, fixed with 2.0% paraformaldehyde, permeabilized with 0.1% saponin in PBS containing 0.5% BSA, then stained with anti-TNFα Ab and subjected to flow cytometry. Only GFAP^+^ HSCs were gated to measure TNFα expression. Black histogram-isotype control; dashed line- unstimulated HSCs; solid line -LPS-stimulated HSCs.
(TIF)

Table S1 **Primers used in qPCR.**
(DOC)

Table S2 **The cells show the number of genes increased, then the number decreased, by LPS stimulation.** There are 1,177 responsive genes exclusive to experiment 1 (shaded bottom data row), 281 exclusive to experiment 2 (shaded right-hand data column) and 576 changes concordant between experiments (dotted box; 345 increases and 252 decreases). Concordant changes therefore represent 33% and 67% of the total changes in experiment 1 and experiment 2 respectively. Of the 1,177 genes exclusive to experiment 1 (i.e., nonresponsive at T1 or T24 in experiment 2) a total of 330 had showed responses at one or more intermediate (3, 6 or 12h) time-points. Of the 281 genes exclusive to experiment 2, 26 showed one or more responses at the intermediate time points in experiment 1.
(DOC)

Table S3 **Values are the significance (as -log(**p**)) of the enrichment of different pathways in different groups.** Pathways were chosen where at least one group had a value > 3 (i.e., p < 0.001). Values < 1.3 (i.e., p > 0.05) were omitted for clarity. The enrichment values in this Table and Table C are calculated by parsing 367 unique genes. This Table contains pathways for which enrichment of the concordant group is greater than that of the aggregate group.
(DOC)

Table S4 **Values are the significance (as -log(**p**)) of the enrichment of different pathways in different groups.** Pathways were chosen where at least one group had a value > 3 (i.e., p < 0.001). Values < 1.3 (i.e., p > 0.05) were omitted for clarity. This Table contains pathways for which enrichment of the aggregate group is greater than that of the concordant group.
(DOC)
