# Supplementary figures and images for: The Transcriptomic Response of Rat Hepatic Stellate Cells to Endotoxin: Implications for Hepatic Inflammation and Immune Regulation
Source: PLoS One. 2013 Dec 9;8(12):e82159. doi: 10.1371/journal.pone.0082159 (PMC3857241; doi:10.1371/journal.pone.0082159)

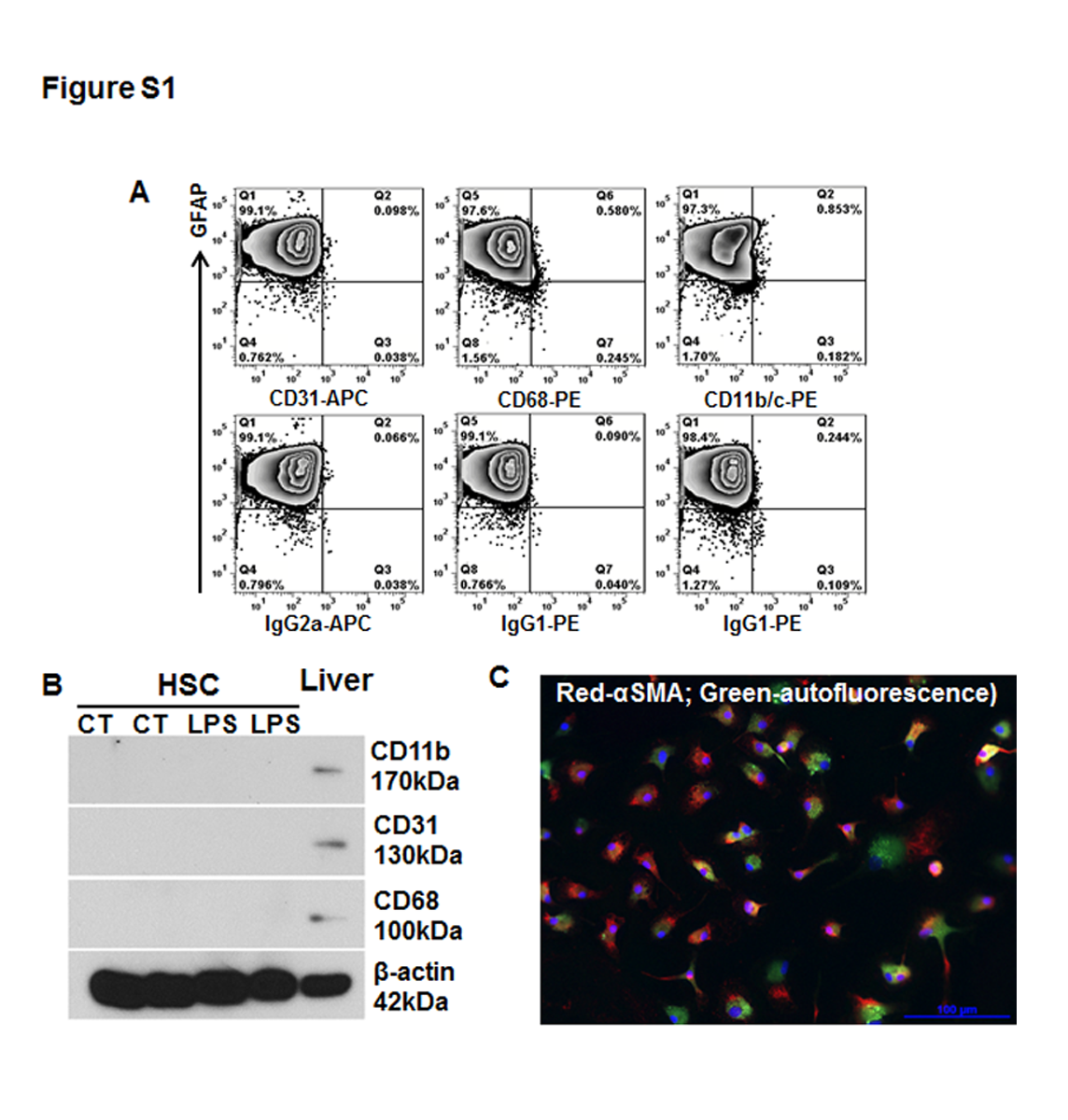

Supplement: Figure S1 — Purity of HSCs. (A) Unstimulated or LPS-stimulated HSCs were harvested using trypsin/EDTA solution, then stained with anti-CD31 (endothelial cell marker), anti-CD68 (Kupffer cell marker), anti-CD11b/c (myeloid cell marker) or anti-GFAP (HSC marker) Abs and subjected to FACS analysis on a LSR II Flow Cytometer. Upper panel shows the purity of HSCs (gated on total live cells), while lower panel shows the respective isotype controls. (B) Protein lysates of unstimulated (CT) or LPS-stimulated (LPS) HSCs were subjected to SDS-PAGE. Separated proteins were transferred on to PVDF membrane and immunoblotted with anti-CD11b , -CD31 or -CD68 Abs. After washing, the membranes were incubated with secondary Ab, and signals were detected using ECL Western blotting detection reagent (GE Healthcare/Amersham, Buckinghamshire, UK). Liver lysates were used for positive control. (C) HSCs on glass coverslips were fixed (with 2% paraformaldehyde), permeabilized and stained for α-SMA (red) and nuclear stain (DAPI). All the cells are stained positive for α-SMA and also contained vitamin A (green autofluorescence). (TIF) [file pone.0082159.s001.tif]

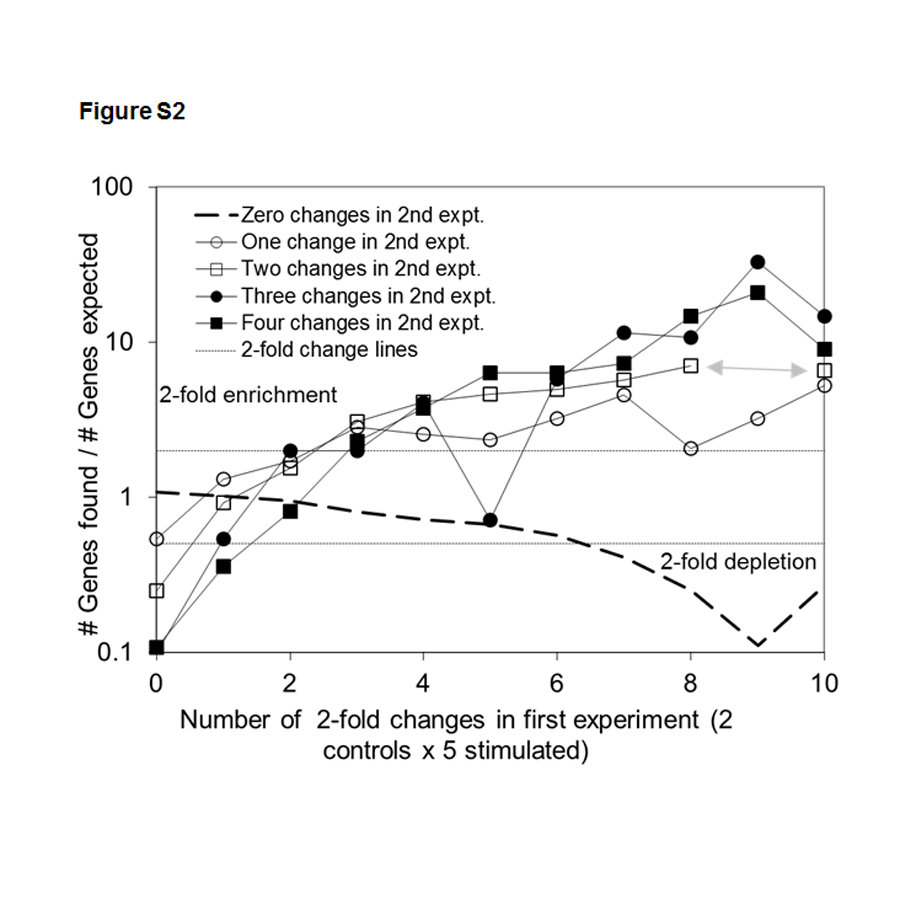

Supplement: Figure S2 — Pairwise comparisons of control vs LPS-stimulated stellate cells. A contingency analysis of the agreement between the first and second experiments shows that genes with ≥3 of 10 valid 2-fold changes in the first experiment are more than twice as likely than chance would predict to be modulated in the second experiment. This concordance is so marked that for ≥ 7 changes in the first experiment there are ≥ 9-fold more genes with ≥ 3 changes than expected in the second experiment, and ≥2-fold fewer genes than expected with zero changes. This is a way of including concordance between genes which are modulated in the second experiment (where 1h and 24h are examined) with genes which respond in the first experiment at intermediate times (3h, 6h or 12h). Exceptions in the Graph: there is no enrichment for 5 changes in experiment 1 and 3 changes in experiment 2; and there are no genes which show 9 changes in experiment 1 and 2 changes in experiment 2 (gap indicated by grey doubled-headed arrow). (TIF) [file pone.0082159.s002.tif]

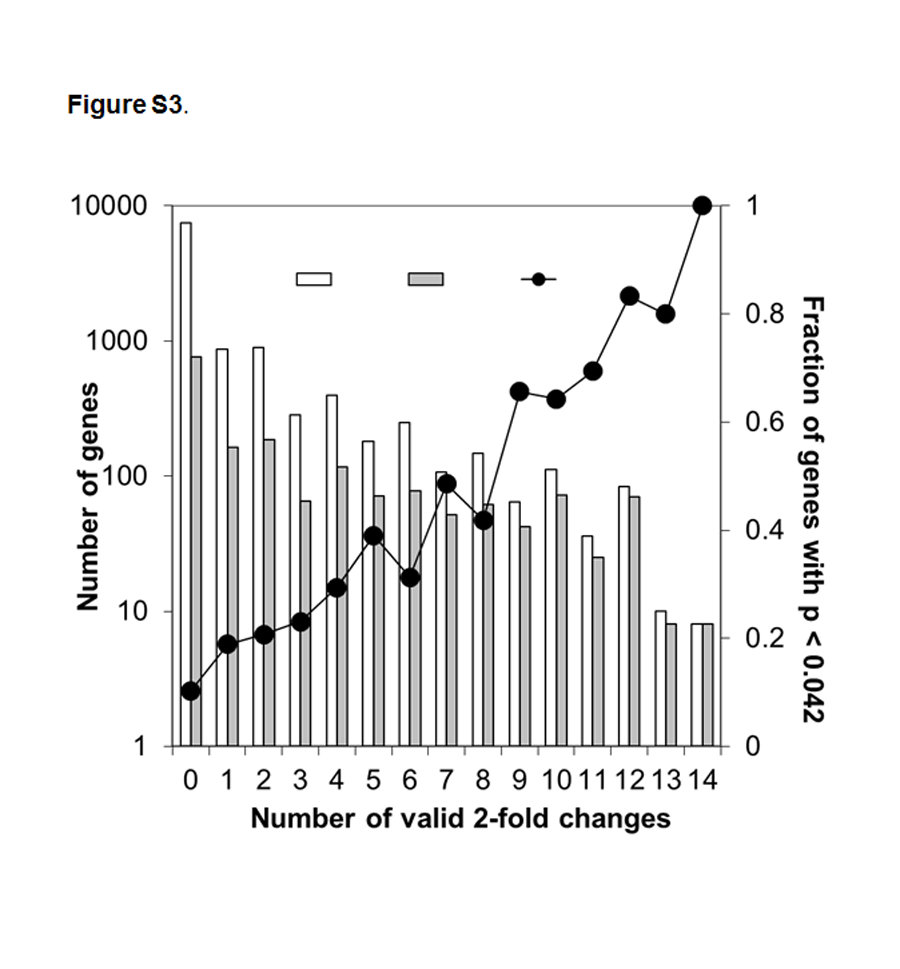

Supplement: Figure S3 — Pairwise comparisons of all control vs stimulated comparisons within each experiment. For experiment 1 this yields (2 control x 5 stimulated = 10 comparisons), while experiment 2 yields (2 control x 2 stimulated = 4 comparisons), for a total of 14 pairwise comparisons. The 10,903 unique characterized genes which shown (14 ≥ n ≥ 0) valid 2-fold differences are shown (unfilled bars). 1,692 unique characterized genes were found with values distributed so that p < 0.042 by the Mann-Whitney test. The numbers of these genes which have corresponding two-fold changes also are shown (filled bars); the fraction they represent of those corresponding changes is shown (filled circles, right hand axis). The progressive increase of this fraction is consistent with a predominant directionality in the modulated genes; i.e. most genes are consistently upregulated or downregulated. (TIF) [file pone.0082159.s003.tif]

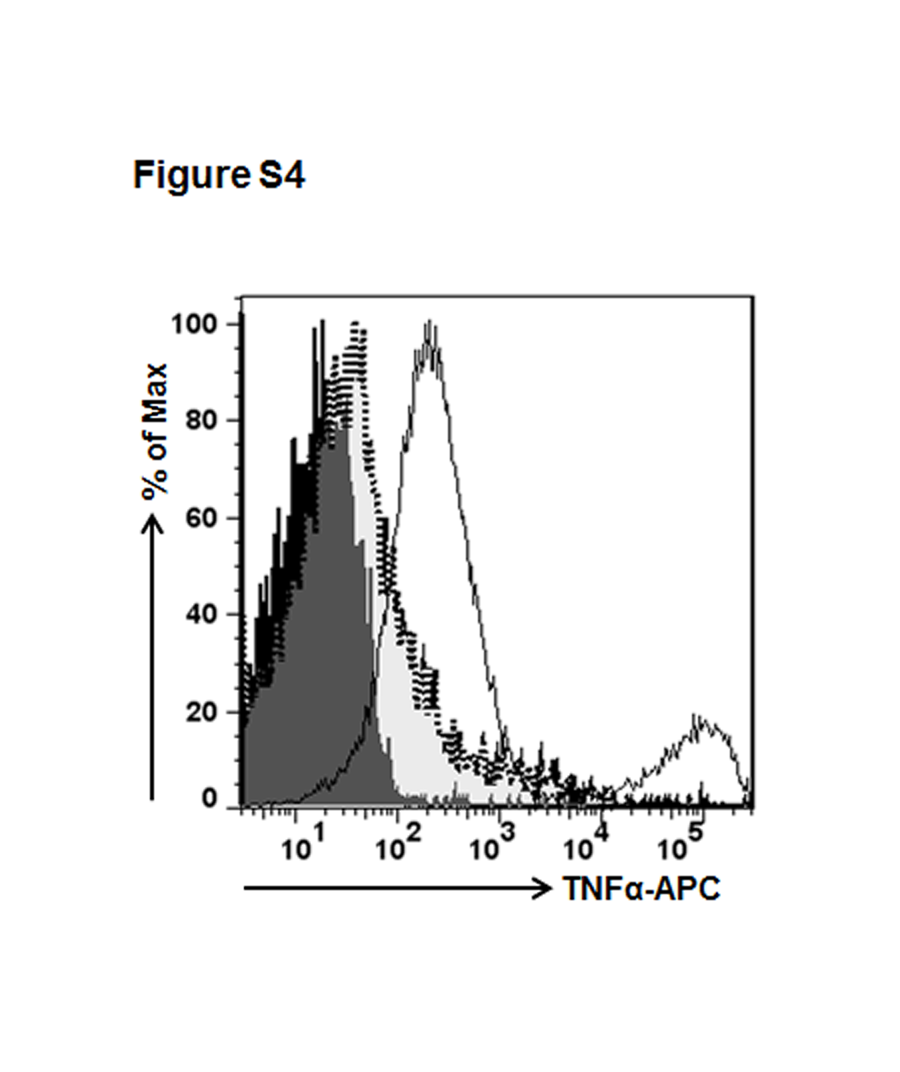

Supplement: Figure S4 — Intracellular staining of TNFα in stellate cells. Unstimulated or LPS-stimulated HSCs were harvested using trypsin/EDTA solution, fixed with 2.0% paraformaldehyde, permeabilized with 0.1% saponin in PBS containing 0.5% BSA, then stained with anti-TNFα Ab and subjected to flow cytometry. Only GFAP+ HSCs were gated to measure TNFα expression. Black histogram-isotype control; dashed line- unstimulated HSCs; solid line -LPS-stimulated HSCs. (TIF) [file pone.0082159.s004.tif]
