# Supplementary material for: The Transcriptomic Response of Rat Hepatic Stellate Cells to Endotoxin: Implications for Hepatic Inflammation and Immune Regulation
Source: PLoS One. 2013 Dec 9;8(12):e82159. doi: 10.1371/journal.pone.0082159 (PMC3857241; doi:10.1371/journal.pone.0082159)
Supplement: Table S1 — Primers used in qPCR. (DOC) [file pone.0082159.s005.doc]

**Table S1:**

| Primer | Sequence |
| --- | --- |
| TLR2 | Forward: CCA AGA GGA AGC CCA AGA AA |
|  | Reverse: CAT GAG GTT CTC CAC CCA ATA G |
| TLR4 | Forward: CGC TCT GGC ATC ATC TTC AT |
|  | Reverse: CGA GGT AGG TGT TTC TGC TAA G |
| TLR7 | Forward: TCC TTG AGT GGC CTA CAA ATC |
|  | Reverse: CTT CAG AGA GCT AGA CTG TTT CC |
| TLR9 | Forward: GGT GAC TAT CAA GCC AGA GAT G |
|  | Reverse: CAG GAA CTG AGA GCC ATT GA |
| IRAK1 | Forward: GAG GCA CTA CCA GAG AAT CAA G |
|  | Reverse: ATG ACA GCA GGG ATG AAC TG |
| IRAK2 | Forward: TTG TAT TGG CCG AGG TTC TC |
|  | Reverse: GGA GTG GAC TGA GGA AGT ATT G |
| IRAK3 | Forward: GTG TCC TTC TCC TCT GTT CTT G |
|  | Reverse: TCC TTG GGA GGT ACT GGA TAG |
| STAT2 | Forward: CTG TAG AGA CTG AGG GCT ATC T |
|  | Reverse: CTA GAG TGT CAG GGT CAC TTT G |
| SOCS3 | Forward: CTA CCC TCC AGC ATC TTT GTC |
|  | Reverse: CAT CAT ACT GGT CCA GGA ACT C |
| TGFβ1 | Forward: GCA ACA ATT CCT GGC GTT AC |
|  | Reverse: GTA TTC CGT CTC CTT GGT TCA G |
| TGFβ-R1 | Forward: ACC GCG TAC CAA ATG AAG AG |
|  | Reverse: CCA GAC CCT GAT GTT GTC ATA TC |
| BAMBI | Forward: CCA CTC CAG CTA CAT CTT CAT C |
|  | Reverse: TAG CAT CTG ATC TCG CCT TTG |
| α-SMA | Forward: AGG GAG TGA TGG TTG GAA TG |
|  | Reverse: GGT GAT GAT GCC GTG TTC TA |
| Collagen-1α | Forward: CAA GAT GGT GGC CGT TAC TAC |
|  | Reverse: GCT GCG GAT GTT CTC AAT CT |
| Fibronectin | Forward: CCA AGT ACA TTC TCA GGT GGA G |
|  | Reverse: GGT CAG GCC TTT GAT GGT ATA G |
| IRF1 | Forward: CTC ACC AAG AAC CAG AGG AAA G |
|  | Reverse: AGA TAA GGT GTC AGG GCT AGA A |
| IRF2 | Forward: GGA AAG CAT CAA CCA GGA GTA G |
|  | Reverse: CTG TCC TTC ACT TCC TCA ATG T |
| IRF7 | Forward: GGC CTA GGA ATC CAC ACT TAA A |
|  | Reverse: CAA GTG TGA CCC AGG TAT TAG G |
| CCL2 | Forward: CAC CTG CTG CTA CTC ATT CA |
|  | Reverse: TAC TAC AGC TTC TTT GGG ACA C |
| CCL4 | Forward: CAC TTC CTG CTG CTT CTC TT |
|  | Reverse: GGC TGC TGG TCT CAT AGT AAT C |
| CCL22 | Forward: CAG GCA CAG TGG CAT AGT TA |
|  | Reverse: CAG GGA AGC AAG AGT GAG TT |
| CXCL1 | Forward: GCA CCC AAA CCG AAG TCA TA |
|  | Reverse: GGG ACA CCC TTT AGC ATC TTT |
| CXCL13 | Forward: CCA AGC TCC AGT GAG TAA GAA A |
|  | Reverse: AAG ATT CCG AGC AGG GAT TAA G |
| CXCL16 | Forward: GCC TCC TTA CAC ACT CAC TAA A |
|  | Reverse: CTA GGA CTG TCA AGG CTG AAT AG |
| Decorin | Forward: CAA GGT CTG CCC ACT TCT ATC |
|  | Reverse: AGC TCA AAC CCA GCT TAG AC |
