# Supplementary material for: The Transcriptomic Response of Rat Hepatic Stellate Cells to Endotoxin: Implications for Hepatic Inflammation and Immune Regulation
Source: PLoS One. 2013 Dec 9;8(12):e82159. doi: 10.1371/journal.pone.0082159 (PMC3857241; doi:10.1371/journal.pone.0082159)
Supplement: Table S2 — The cells show the number of genes increased, then the number decreased, by LPS stimulation. There are 1,177 responsive genes exclusive to experiment 1 (shaded bottom data row), 281 exclusive to experiment 2 (shaded right-hand data column) and 576 changes concordant between experiments (dotted box; 345 increases and 252 decreases). Concordant changes therefore represent 33% and 67% of the total changes in experiment 1 and experiment 2 respectively. Of the 1,177 genes exclusive to experiment 1 (i.e., nonresponsive at T1 or T24 in experiment 2) a total of 330 had showed responses at one or more intermediate (3, 6 or 12h) time-points. Of the 281 genes exclusive to experiment 2, 26 showed one or more responses at the intermediate time points in experiment 1. (DOC) [file pone.0082159.s006.doc]

**Table S2:**

| Experiment 1: | Response at both T1 and T24 | Response at T1 only | Response at T24 only | No response |
| --- | --- | --- | --- | --- |
|  |  | | | |
| Experiment 2: |  |  |  |  |
| Response at both T1 and T24 | 18, 0 | 10, 1 | 134, 106 | 11, 51 |
| Response at T1 only | 0, 0 | 0, 0 | 26, 9 | 40, 23 |
| Response at T24 only | 0, 0 | 1, 0 | 138, 133 | 45, 111 |
| No response | 18, 3 | 73, 6 | 362, 715 | 5,486 |
| Exp1 response at other timepoints | 5, 0 | 5, 4 | 83, 233 |  |
