# Supplementary material for: The Transcriptomic Response of Rat Hepatic Stellate Cells to Endotoxin: Implications for Hepatic Inflammation and Immune Regulation
Source: PLoS One. 2013 Dec 9;8(12):e82159. doi: 10.1371/journal.pone.0082159 (PMC3857241; doi:10.1371/journal.pone.0082159)
Supplement: Table S3 — Values are the significance (as -log(p)) of the enrichment of different pathways in different groups. Pathways were chosen where at least one group had a value > 3 (i.e., p < 0.001). Values < 1.3 (i.e., p > 0.05) were omitted for clarity. The enrichment values in this Table and Table C are calculated by parsing 367 unique genes. This Table contains pathways for which enrichment of the concordant group is greater than that of the aggregate group. (DOC) [file pone.0082159.s007.doc]

**Table S3:**

| Pathway | Aggregate | Exp 1 only | Concordant | Exp 2 only |
| --- | --- | --- | --- | --- |
| Communication between Innate and Adaptive Immune Cells | 11.6 |  | 12.3 | 2.7 |
| TREM1 Signaling | 8.61 |  | 11.5 |  |
| Atherosclerosis Signaling | 6.23 |  | 10.1 |  |
| LXR/RXR Activation | 4.25 |  | 9.23 |  |
| Differential Regulation of Cytokine Production in Intestinal Epithelial Cells by IL-17A and IL-17F | 6.2 |  | 8.57 |  |
| Differential Regulation of Cytokine Production in Macrophages and T Helper Cells by IL-17A and IL-17F | 5.99 |  | 7.78 |  |
| Acute Phase Response Signaling | 3.74 |  | 7.47 |  |
| Role of Pattern Recognition Receptors in Recognition of Bacteria and Viruses | 6.52 |  | 7.25 |  |
| Dendritic Cell Maturation | 6.75 |  | 6.77 | 2.54 |
| IL-6 Signaling | 4.98 |  | 6.46 |  |
| Role of IL-17F in Allergic Inflammatory Airway Diseases | 3.26 |  | 6.4 |  |
| Role of Cytokines in Mediating Communication between Immune Cells | 3.82 |  | 5.96 |  |
| Role of IL-17A in Arthritis | 3.42 |  | 5.58 |  |
| Activation of IRF by Cytosolic Pattern Recognition Receptors | 4.41 |  | 5.47 |  |
| IL-10 Signaling | 5.11 |  | 5.27 |  |
| Role of Macrophages, Fibroblasts and Endothelial Cells in Rheumatoid Arthritis | 4.67 |  | 5.08 | 1.4 |
| Hepatic Cholestasis | 2.16 |  | 4.76 |  |
| Colorectal Cancer Metastasis Signaling | 3.26 |  | 4.64 |  |
| NF-κB Signaling | 3.28 |  | 4.21 |  |
| IL-17 Signaling | 2.47 |  | 4.07 |  |
| PPAR Signaling | 2.29 |  | 3.72 |  |
| Role of IL-17A in Psoriasis | 2.55 |  | 3.61 |  |
| DNA damage-induced 14-3-3Ïƒ Signaling |  |  | 3.43 |  |
| Chondroitin Sulfate Degradation (Metazoa) | 2.25 |  | 3.37 |  |
| Systemic Lupus Erythematosus Signaling |  |  | 3.34 |  |
| Toll-like Receptor Signaling | 2.18 |  | 3.32 |  |
| IL-17A Signaling in Airway Cells |  |  | 3.28 |  |
| HMGB1 Signaling | 1.66 |  | 3.12 |  |
| Epithelial Adherens Junction Signaling | 1.69 | **3.28** |  |  |
