# Supplementary material for: The Transcriptomic Response of Rat Hepatic Stellate Cells to Endotoxin: Implications for Hepatic Inflammation and Immune Regulation
Source: PLoS One. 2013 Dec 9;8(12):e82159. doi: 10.1371/journal.pone.0082159 (PMC3857241; doi:10.1371/journal.pone.0082159)
Supplement: Table S4 — Values are the significance (as -log(p)) of the enrichment of different pathways in different groups. Pathways were chosen where at least one group had a value > 3 (i.e., p < 0.001). Values < 1.3 (i.e., p > 0.05) were omitted for clarity. This Table contains pathways for which enrichment of the aggregate group is greater than that of the concordant group. (DOC) [file pone.0082159.s008.doc]

**Table S4:**

| Pathway | Aggregate | Exp 1 only | Concordant | Exp 2 only |
| --- | --- | --- | --- | --- |
| Granulocyte Adhesion and Diapedesis | 18.9 | 1.68 | 15.1 | 3.31 |
| Agranulocyte Adhesion and Diapedesis | 15.5 | 2.8 | 10.3 | 1.97 |
| Hepatic Fibrosis / Hepatic Stellate Cell Activation | 14.4 | 4.56 | 8.05 |  |
| Altered T Cell and B Cell Signaling in Rheumatoid Arthritis | 8.63 |  | 7.96 | 4.09 |
| Crosstalk between Dendritic Cells and Natural Killer Cells | 8.19 |  | 6.84 | 2.34 |
| Graft-versus-Host Disease Signaling | 8.06 |  | 6.55 | 3.69 |
| Complement System | 6.52 | 1.6 | 5.66 |  |
| Role of Hypercytokinemia/hyperchemokinemia in the Pathogenesis of Influenza | 6.3 |  | 5.91 |  |
| Antigen Presentation Pathway | 6.23 |  | 4.34 | 3.06 |
| T Helper Cell Differentiation | 5.62 |  | 3.55 | 3.49 |
| Allograft Rejection Signaling | 5.48 |  | 3.35 | 2.51 |
| OX40 Signaling Pathway | 5.26 |  | 2.74 | 2.15 |
| Interferon Signaling | 5.1 |  | 3.85 |  |
| Pathogenesis of Multiple Sclerosis | 4.93 |  | 3.61 |  |
| p38 MAPK Signaling | 4.79 | 2.07 | 2.57 |  |
| Inhibition of Matrix Metalloproteases | 4.64 | 2.08 | 2.81 |  |
| Role of Osteoblasts, Osteoclasts and Chondrocytes in Rheumatoid Arthritis | 4.13 |  | 3.03 |  |
| HIF1α Signaling | 4.1 | 2.16 | 2.65 |  |
| Cytotoxic T Lymphocyte-mediated Apoptosis of Target Cells | 4.01 |  | 2.31 | **2.32** |
| Caveolar-mediated Endocytosis Signaling | 4 | **3.27** |  |  |
| iNOS Signaling | 3.98 | 1.35 | 2.74 |  |
| IL-8 Signaling | 3.94 | 1.78 | 2.41 |  |
| GADD45 Signaling | 3.81 | 1.41 | 2.32 |  |
| Type I Diabetes Mellitus Signaling | 3.77 |  | 3.23 | 2.14 |
| NF-κB Activation by Viruses | 3.68 |  | 2.16 |  |
| Role of Tissue Factor in Cancer | 3.65 |  | 2.46 |  |
| Leukocyte Extravasation Signaling | 3.61 | **2.21** | 1.57 |  |
| B Cell Development | 3.49 |  | 1.65 | **2.66** |
| Autoimmune Thyroid Disease Signaling | 3.45 |  | 2.13 | **2.73** |
| Aryl Hydrocarbon Receptor Signaling | 3.42 |  | 2.95 |  |
| Death Receptor Signaling | 3.29 |  | 2.44 |  |
| Virus Entry via Endocytic Pathways | 3.23 | **2.67** |  |  |
| Cell Cycle: G2/M DNA Damage Checkpoint Regulation | 3.12 | 1.46 | 1.58 |  |
